# Tailoring innovative silver nanoparticles for modern medicine: The importance of size and shape control and functional modifications

**DOI:** 10.1016/j.mtbio.2025.102071

**Published:** 2025-07-09

**Authors:** Ibtissam Laib, Noura Gheraissa, Abir Benaissa, Latra Benkhira, Manel Azzi, Yousef Benaissa, Ahmed G. Abdelaziz, Furong Tian, Maureen Walsh, Mikhael Bechelany, Ahmed Barhoum

**Affiliations:** a Department of Cellular and Molecular Biology, El Oued University, Algeria; b Laboratory of Biodiversity and Biotechnology Applications in Agriculture, University of El Oued, 39000, Algeria; c Laboratory of Biotechnology Biomaterial and Condensed Matter, Department of Process Engineering and Petrochemistry, Faculty of Technology, University of El Oued, El-Oued, 39000, Algeria; d Department of Process Engineering and Petrochemistry, Faculty of Technology, University of El Oued, 789, 39000, El Oued, BP, Algeria; e Renewable Energy Development Research Unit in Arid Zones (UDERZA), University of El Oued, BP789, 39000, El Oued, Algeria; f Laboratory of Biology, Environment and Health, Faculty of Natural and Life Sciences, University of El Oued, 39000, Algeria; g VPRS Laboratory, Chemistry Department, Faculty of Mathematics and Matter Sciences. University of KASDI Merbah, Ouargla, 30000, Algeria; h Biochemistry Division, Chemistry Department, Faculty of Science, Helwan University, Cairo, 11795, Egypt; i Nanolab Research Centre, Physical to Life Sciences Research Hub, Technological University Dublin, Camden Row, D08 CKP1, Dublin, Ireland; j Chemical and BioPharmaceutical Sciences, Technological University Dublin, Tallaght Campus, 24, Dublin, D24 FKT9, Ireland; k Institut Européen des Membranes, IEM, UMR-5635, University Montpellier, ENSCM, CNRS, Place Eugène Bataillon, 34095, Montpellier, France; l Functional Materials Group, Gulf University for Science and Technology, Mubarak Al-Abdullah, 32093, Kuwait; m Chemical and BioPharmaceutical Sciences, Technological University Dublin, Grangegorman Campus, 7, Dublin, D07 ADY7, Ireland

**Keywords:** Silver nanoparticles, Green synthesis, Surface functionalization, Regenerative medicine, Cancer immunotherapy, Antimicrobial resistance, Nanomedicine, Gene therapy

## Abstract

Silver nanoparticles (Ag NPs) play a significant role in modern medicine, with their size, shape, and surface chemistry strongly influencing their biological performance. Smaller nanoparticles (1–100 nm) can penetrate cells more easily, while different shapes such as spheres, rods, cubes, triangles, wires, and stars impact their antimicrobial activity and drug delivery efficiency. This review highlights the importance of precise control over these properties during synthesis to optimize their therapeutic potential. Green synthesis methods, including plant-based and microbial approaches, are presented as safer, more cost-effective, and environmentally friendly alternatives to conventional chemical processes. The review also discusses surface functionalization strategies such as biodegradable coatings, targeting ligands, and hybrid structures that enhance nanoparticle stability, reduce toxicity, and enable selective delivery to sites like tumors or infected tissues. As highlighted in the review, Ag NPs hold great promise in antimicrobial therapy, targeted drug delivery, diagnostics, tissue engineering, stem cell support, and cancer treatment. Emerging research also indicates potential benefits in modulating inflammation and oxidative stress in neurological and cardiovascular disorders. Despite these advances, key challenges remain, including low synthesis yields, poor reproducibility, scale-up difficulties, potential toxicity, uncertain biological mechanisms, and a lack of regulatory clarity. The review indicates the urgent need for improved synthesis control and rigorous safety evaluations to support the clinical translation of Ag NP-based technologies.

## Highlights

• Green synthesis offers eco-friendly, scalable production of biocompatible Ag NPs.
• Size, shape, and surface functionalization are key to optimizing Ag NP performance.
• Ag NPs show broad biomedical potential—from antimicrobial use to cancer therapy.
• Challenges remain in safety, large-scale production, and clinical translation.

## Graphical abstract

**Figure:**
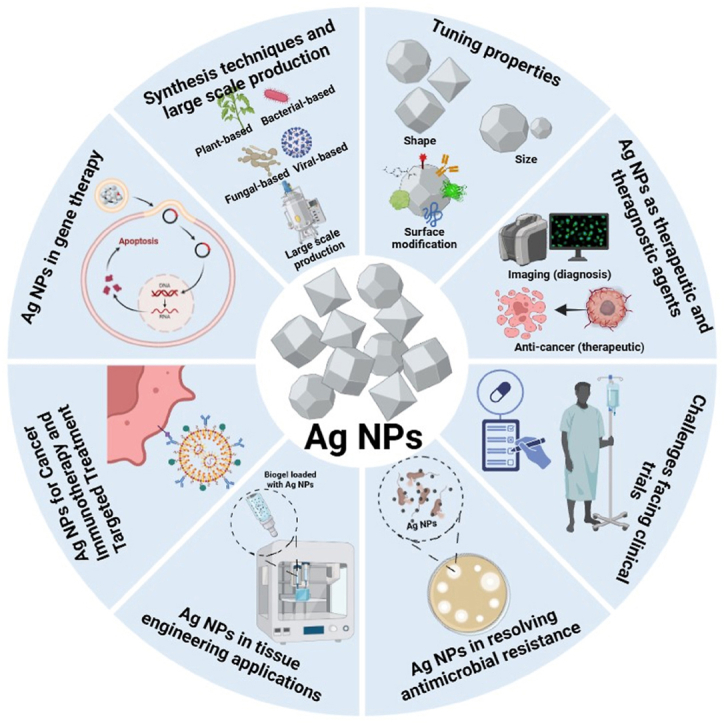

## 1 Introduction

Noble metal (e.g. gold, silver, platinum)-based nanoparticles (NPs) play a vital role in nanomedicine due to their exceptional stability, biocompatibility and unique properties [1]. For example, by absorbing light and converting it into heat, they selectively destroy up to 90 % of tumor cells without damaging non-malignant cells [2]. Moreover, Ag NPs have potent antimicrobial properties [3]. They have been incorporated in wound dressings and medical device coatings to prevent infections and they reduce bacterial contamination by up to 70 % [4]. Ag NPs are also tested in combination therapies, enhancing the effectiveness of classical antibiotics against drug-resistant bacteria [5,6]. In addition, noble metal NPs are valuable in diagnostic applications and have been integrated into imaging techniques, such as computed tomography (CT), to enhance contrast and improve diagnostic accuracy [7,8]. These applications highlight the critical role of noble metal NPs in targeted therapies, infections, and diagnostic tools, paving the way for more effective and personalized healthcare solutions [9].

Ag NPs display interesting properties for applications in nanomedicine, particularly their small size (1–100 nm) that enables them to interact with biological systems at the molecular level, enhancing their biological activity [10,11]. Ag NP antimicrobial, antioxidant and anti-inflammatory properties make them effective against many pathogens, including antibiotic-resistant bacteria, fungi and viruses [12]. Their optical properties, such as localized surface plasmon resonance (LSPR), further enhance their role in biosensing and imaging applications. Silver is among several biologically relevant metal elements, such as zinc, copper, iron, and selenium, that play crucial roles in physiological functions [13]. However, unlike essential trace metals like zinc and copper, silver particularly in the form of Ag NPs is not an essential element and may pose health risks under certain conditions [14]. When used in medical applications, the release of Ag^+^ ions could lead to adverse effects, such as cytotoxicity, inflammation and organ toxicity, especially when Ag NPs accumulate in tissues [15]. Therefore, to ensure their safe use in the clinic, their size, shape and surface functionalization must be precisely controlled [16,17]. For instance, Ag NPs can be functionalized with biocompatible polymers, biodegradable coatings, or targeting ligands to reduce their toxicity and improve their safety profile [[18], [19], [20]]. Additionally, controlled release mechanisms ensure that Ag^+^ ions are gradually released, reducing the risk of toxic buildup. Research on the long-term effects of Ag NPs and their safe dosage is ongoing, and with proper modifications, their therapeutic potential can be harnessed while minimizing health risks [14,21].

Ag NP controlled synthesis also contributes to optimize their biomedical applications and minimize toxicity [22]. Ag NPs can be synthesized using physical, chemical and green approaches. Each method significantly influences their size, shape and surface properties. These synthesis techniques yield diverse morphologies (e.g. spherical, cubic, rod-like, flower-like) that play a crucial role in determining their biomedical efficacy [23,24]. Rod-shaped Ag NPs exhibit enhanced light scattering properties, making them ideal for applications in biosensing and photothermal therapy [25,26]. Triangular and cubic Ag NPs possess unique plasmonic properties that promote light absorption and scattering, contributing to their use in cancer treatment and diagnostic imaging [27,28]. Green synthesis methods, for instance using plant extracts of *Camellia sinensis* (green tea) and *Cinnamomum verum* (cinnamon), allow controlling the particle size (typically 10–30 nm) and shape [29], do not require toxic chemicals, and produce very stable Ag NPs. Surface functionalization further enhances their properties. For instance, coating with polymers, such as polyethylene glycol (PEG), increases the Ag NP biocompatibility, reduces their aggregation, and prolongs their circulation time in the bloodstream. Targeting ligands (e.g. folic acid) can be attached to the Ag NP surface to direct them towards specific cells, such as cancer cells [30]. Additionally, modification with biopolymers (e.g. chitosan or alginate) helps to reduce Ag NP cytotoxicity and supports tissue regeneration. By carefully controlling their size, shape and surface functionalization, Ag NPs can be tailored for specific medical applications in order to optimize their therapeutic efficacy and minimize their harmful side effects [31].

This review explores the potential of Ag NPs in modern medicine. It highlights their tunable size, shape and functional modifications that enhance their biomedical applications. Ag NPs exhibit unique physicochemical properties, making them valuable in antimicrobial resistance, cancer therapy and regenerative medicine. Unlike previous reviews, this study provides a detailed comparison of different green synthesis methods (microbial and plant-based approaches) and discusses large-scale production challenges and emerging solutions. Then, it analyzes advanced functionalization strategies, such as ligand conjugation, polymer coating, and hybrid nanostructures, to improve Ag NP stability, biocompatibility and targeted therapeutic efficiency. Compared with the existing literature, this study provides an in-depth discussion on how these modifications optimize drug delivery, enhance theragnostic applications and reduce toxicity. The integration of multifunctional Ag NPs into bioactive scaffolds, 3D bioprinting, and stem cells is transforming immunotherapy, gene editing and precision medicine. This highlights how Ag NPs can revolutionize modern healthcare, offering sustainable and effective solutions for critical medical challenges.

## 2 Green and biological synthesis techniques

**Fig. 1:**
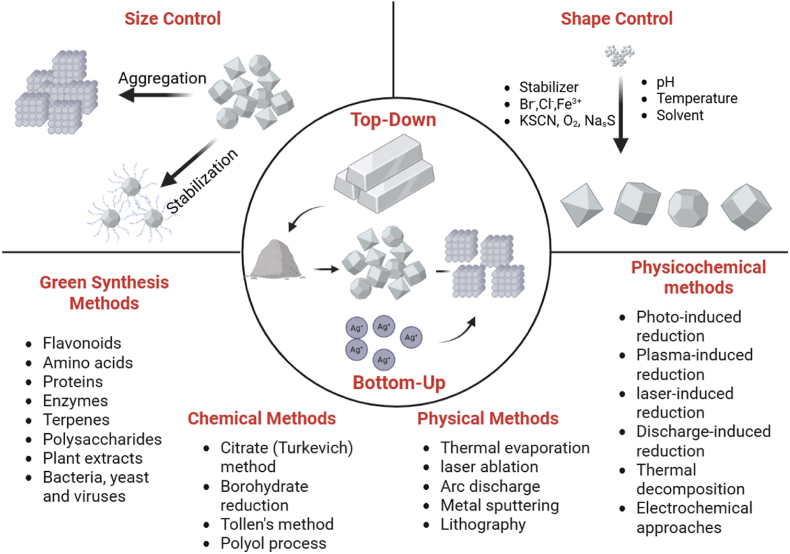
Various methods for producing Ag NPs and the key parameters influencing their size and shape. Created with BioRender.com.

Size and morphology of Ag NPs is strongly influenced by synthesis parameters such as pH, temperature, phytochemical composition, and precursor concentration. It offers better size and shape control compared to other metal nanoparticles such as zinc, copper, and iron, making them highly desirable for sizes and shapes [32]. The pH affects the reduction rate of Ag^+^ ions and particle stability, with higher pH generally producing smaller, more uniform nanoparticles due to faster nucleation. Temperature plays a key role in reaction kinetics. Moderate heating promotes faster nucleation, leading to the formation of smaller and more crystalline particles. In contrast, excessive heating can cause particle aggregation or the development of irregular shapes. Different top-down and bottom-up methods for producing Ag NPs, along with the parameters affecting their size and shape, are illustrated in Fig. 1.

In green synthesis, phytochemicals like flavonoids and phenolics serve as natural reducing and capping agents, guiding particle growth and shape by binding selectively to crystal facets, enabling diverse morphologies such as rods or triangles [33]. Spherical nanoparticles form under isotropic conditions with mild reducers, while anisotropic forms (rods, stars, cubes, triangular plates) arise when specific biomolecules or halides guide facet-selective growth [23]. The concentration of silver precursor ions also impacts size, with higher concentrations promoting larger particles but risking aggregation. Careful optimization of these factors is critical to produce Ag NPs with desired size, shape, and surface properties tailored for specific biomedical and technological applications, ensuring enhanced stability, functionality, and biocompatibility [34].

**Fig. 2:**
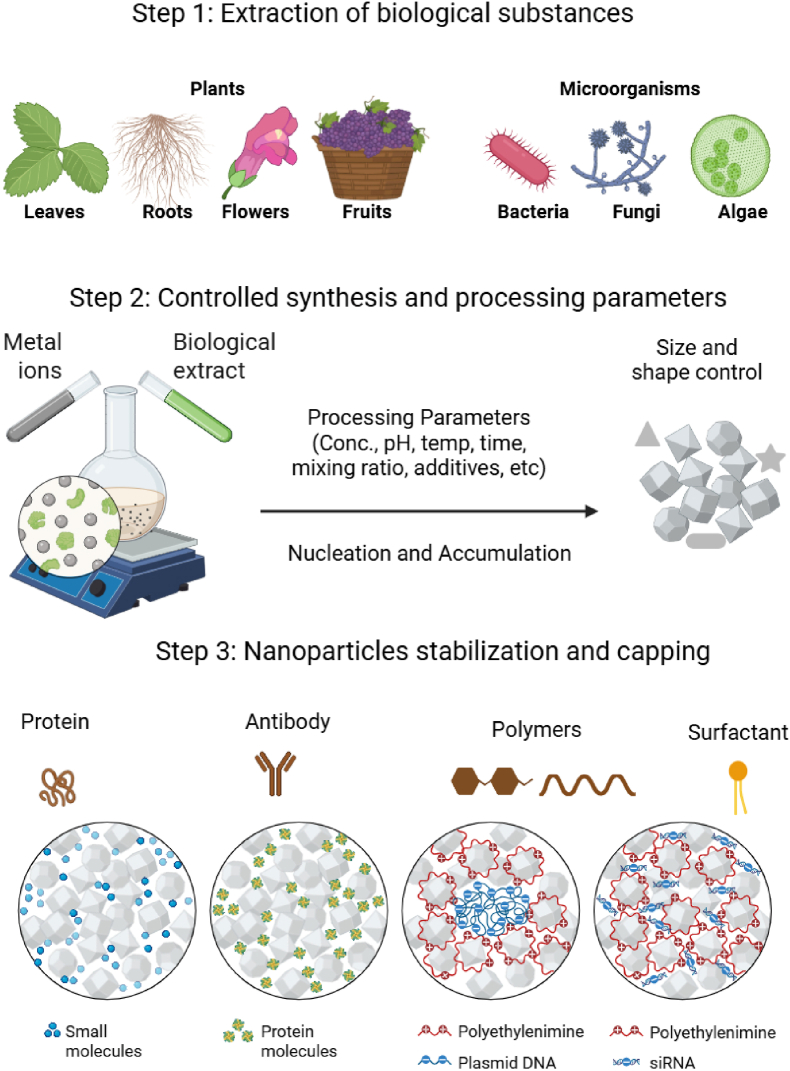
Various green approaches for Ag NP synthesis and their applications; the extracts act as reducing, capping and stabilizing agents. Created with BioRender.com.

Green synthesis has gained significant attention as an eco-friendly and sustainable approach for Ag NP production [32]. Unlike chemical synthesis, which often requires hazardous reducing agents (e.g. sodium borohydride), green synthesis uses reducing and stabilizing agents derived from plants, fungi, algae, bacteria and viruses (Fig. 2) [32]. This minimizes the environmental toxicity and enhances the Ag NP biocompatibility, scalability and functionality for various biomedical and industrial applications [32]. Compared to other metal nanoparticles such as zinc (Zn), copper (Cu), iron (Fe), and gold (Au), silver (Ag) NPs offer better shape and size control under green synthesis conditions due to their favorable nucleation and growth kinetics. Moreover, while Au NPs are expensive and Zn, Cu, and Fe NPs may suffer from oxidation and limited morphology control, Ag NPs show a balance between cost-effectiveness, stability, and tunable morphology, making them a preferred choice in green nanotechnology (see Table 1).

**Table 1:** Biological sources, green synthesis methods, and Ag NP size, shape, properties and applications.

Organism type	Species/Strain	Synthesis method	Ag NP size and shape	Applications/properties	Reference
Plants	*Diospyros malabarica*	Leaf extract	Spherical; 20 nm	Anticancer, antibacterial	[47]
	*Andrographis echioides*	Leaf extract	Cubic, pentagonal, hexagonal; 68.06–91.28 nm	Antibacterial	[48]
	*Andrographis paniculata*	Leaves	Cubic; 40 and 60 nm	Antibacterial	[49]
	*Azadirachta indica*	Leaves	Poly-dispersed; less than 40 nm	Antibacterial	[50]
	*Phyllanthus amarus*	Leaves	Flower-like; 30 nm–42 nm	Antibacterial	[51]
	*Gloriosa superba*	Leaves	Triangular; 20 nm	Antibacterial and antibiofilm	[52]
	*Ocimum tenuiflorum*	Leaves	Cuboidal; 50 nm	Antimicrobial	[53]
	*Syzygium guineense*	Leaf extract	Cylindrical, triangular and prismatic	Antibacterial	[54]
	*Alhagi graecorum*	Leaf extract	Spherical; 22–36 nm	Antifungal, antitumor	[55]
Algae	*Padina* spp.	Marine macroalgae	Spherical; 23 nm	Antibacterial	[56]
Fungi	*Penicillium* spp.	Extracellular/intracellular synthesis	Spherical; 50 nm	Biomedical applications	[57]
	*Fusarium oxysporum*	Extracellular/intracellular synthesis	Spherical; 22–50 nm	Biomedical applications	[36]
Yeast	*MKY3*	Extracellular synthesis	Spherical; 2–5 nm	Biomedical applications	[58]
Bacteria	*Morganella morganii*	Intracellular synthesis	Various shapes	Biomedical applications	[59]
	*Bacillus licheniformis*	Intracellular synthesis	Various shapes	Biomedical applications	[60]
	Lactic acid bacteria	Extracellular synthesis	Spherical or polyhedral; 2–45 nm	Antifungal	[61]
	*Enterococcus* spp.	Intracellular synthesis	Spherical	Pro-apoptotic agent	[62]
Viruses	*Tobacco mosaic virus (TMV)*	Biotemplate	Round-shaped; 3.37 ± 12.7 nm	Electrical conductivity, biomedical applications	[63]

Plant-based synthesis is a widely accessible and cost-effective method in which phytochemicals, such as polyphenols, flavonoids, and terpenoids, are used to mediate nanoparticle formation [33]. Ag NPs synthesized using *Azadirachta indica* (neem) leaf extracts typically exhibit a cubic face-centered morphology with sizes <30 nm and enhanced antimicrobial activity compared with chemically synthesized Ag NPs [33]. However, the phytochemical composition of the extracts varies in function of the plant species, extraction methods and growth conditions, affecting reproducibility. Moreover, plant-based synthesis is simpler and more cost-effective than other biological methods, but offers less precise control over the NP size and shape [34,35].

Fungal-mediated synthesis is another effective method for Ag NP production. For instance, *Fusarium oxysporum* and *Ganoderma lucidum* provide reducing and capping agents through metabolites, such as enzymes and proteins, resulting in Ag NPs with high stability and uniform size distribution [36]. Ag NPs synthesized using *F. oxysporum* range from 25 nm to 50 nm in size and exhibit excellent antimicrobial and anticancer properties and low toxicity. Fungal-mediated synthesis is advantageous for large-scale production due to the high yields in bioreactors. Additionally, fungi can be grown on agro-industrial waste, promoting the circular economy [37]. However, fungal-mediated synthesis is slower compared to bacterial-based methods and may involve complex culturing conditions that might limit scalability [37].

Recent studies have reinforced the value of fungal-based synthesis as a safe and effective route for producing bioactive Ag NPs. Mehrdel et al. (2023) [38] reported the successful mycosynthesis of phenol-capped Ag NPs (∼11.8 nm) using Agaricus bisporus, exhibiting excellent colloidal stability and potent antibacterial activity against Pseudomonas aeruginosa and Streptococcus pyogenes, with no observed *in vivo* toxicity supporting their use in topical biomedical products. Likewise, Owaid et al. (2021) [39] introduced *Picoa* sp. (Pezizales) as a novel fungal template for Ag NP production, generating particles with high stability (−20.9 mV) and strong antifungal activity, highlighting their biosafety and agricultural potential. Dheyab et al. (2025) [40] reviewed the advantages of green synthesis using biological agents, emphasizing improved biocompatibility, reduced toxicity, and broad-spectrum therapeutic applications of green-synthesized Ag NPs. Additionally, Jameel et al. (2022) [41] demonstrated that ultrasound-assisted extraction of phenolics from A. bisporus enabled the fabrication of uniform cubic Ag NPs (∼50 nm) with enhanced antibacterial properties. Collectively, these studies underscore the potential of fungal- and plant-based synthesis to generate safe, stable, and functionally optimized Ag NPs for clinical and environmental applications.

Microalgae (e.g., *Spirulina platensis*, *Chlorella vulgaris*) and macroalgae (e.g., *Sargassum*, *Gracilaria*) are eco-friendly platforms for Ag NP synthesis [42]. Algae-derived NPs exhibit excellent stability, biocompatibility and antimicrobial properties, which are ideal for pharmaceutical applications. Moreover, algae can grow in wastewater, reducing production costs and contributing to environmental remediation [42]. However, the optimization of growth conditions and harvesting methods can increase complexity and production times compared with bacterial-mediated synthesis. Moreover, their scalability and efficiency are still limited compared with fungal- and bacterial-based synthesis methods [42].

Bacterial-mediated synthesis, for instance using *Pseudomonas aeruginosa*, *Bacillus subtilis* or *Escherichia coli*, is a rapid, cost-effective and ecofriendly method for Ag NP production [43]. *P. aeruginosa* produces monodisperse Ag NPs with antimicrobial activity and reduced toxicity compared with chemical synthesis [43]. Bacterial synthesis outperforms fungal- and plant-based methods in terms of speed and scalability, but the potential pathogenicity of some bacterial strains and the requirement of sterile conditions can limit their application [43].

Viral-mediated synthesis, for instance using M13 bacteriophages and T7 phages, allows precisely controlling the NP morphology and size [44]. Viral templates bind to Ag^+^ ions via surface proteins, facilitating the formation of monodisperse Ag NPs with high stability and biocompatibility. Nevertheless viral synthesis is more complex and costly compared with other green methods, limiting its use to specific applications, such as drug delivery and bioimaging [44].

Green-synthesis of Ag NPs displays significant advantages, particularly environmental sustainability, reduced toxicity and enhanced biocompatibility, compared with the traditional chemical methods [45]. Green synthesis does not require toxic reagents, thereby reducing environmental pollution and minimizing energy consumption. Moreover, the synthesized Ag NPs are less toxic and more biocompatible, making them suitable for medical and biotechnology applications [45,46].

### 2.1 Tuning Ag NP size, shape and surface modification for enhanced properties

**Fig. 3:**
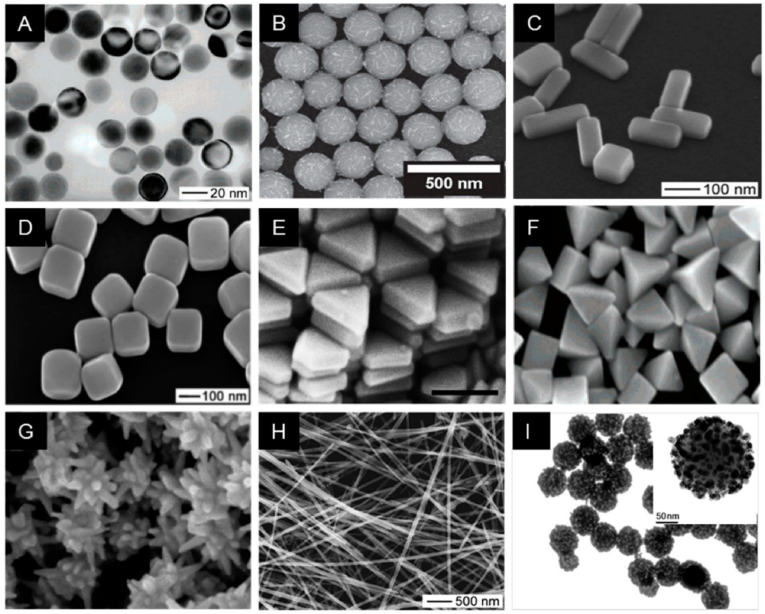
Electron microscopy images of Ag nanostructures with various sizes and morphologies: (A) Nanospheres, (B) Necklaces, (C) Nanobars, (D) Nanocubes, (E) Nanoprisms, (F) Bipyramids, (G) Nanostars, (H) Nanowires, and (I) Ag NP-embedded silica particles [10]. Open Access, MDPI.

The size, shape, and surface modifications of Ag NPs strongly influence their interactions, stability and effectiveness as antimicrobial and anticancer agents and catalysts [64]. Ag NPs can be synthesized in various shapes (spherical, cubic, prismatic, bipyramidal, star-like, wire-like) that display distinct properties (Fig. 3). For instance, spherical and cubic Ag NPs enhance antimicrobial activity, while star-shaped and prismatic Ag NPs have superior optical properties for biosensing.

Smaller Ag NPs (<10 nm) have higher antimicrobial activity due to their increased surface area and greater Ag^+^ ion release [65]. For example, small (5–15 nm) spherical Ag NPs synthesized using sodium borohydride show strong antibacterial effects against *E. coli* and *Staphylococcus aureus* by disrupting bacterial membranes and generating reactive oxygen species (ROS) [66]. Cubic Ag NPs (20–50 nm, green synthesis using *A. indica*) exhibit excellent antibacterial properties due to the higher surface energy that facilitate bacterial attachment and interaction [67]. Moreover, compared with bare spherical Ag NPs (67.17 nm, sodium borohydride-based synthesis), bovine serum albumin-coated spherical Ag NPs (20–45 nm) exhibit lower cytotoxicity and improved biocompatibility and similar antibacterial efficacy [68]. Rod-shaped Ag NPs (30–60 nm, synthesized using a *Moringa oleifera* extract) show increased cytotoxicity toward cancer cells because they can penetrate cell membranes more effectively than spherical Ag NPs [69]. On the other, star-shaped Ag NPs (60–80 nm, citrate reduction method) display enhanced plasmonic and catalytic activity, making them ideal for biosensor and photothermal therapy applications [70].

Surface modifications with polymers, biomolecules, or surfactants improve the stability, dispersibility and biocompatibility of Ag NPs, broadening their applications in drug delivery, wound healing and environmental remediation. Surface modification with chitosan improves the Ag NP biocompatibility and reduces toxicity towards mammalian cells, without affecting their antimicrobial activity against multidrug-resistant bacteria [71]. Similarly, PEG-coating of Ag NPs (3–10 nm, spherical) increases their circulation time in the bloodstream and reduces opsonization, making them suitable for drug delivery applications [72].

Ag NPs behave differently in biological environments like blood, tissue fluids, or tumor sites due to factors such as protein corona formation, pH, ionic strength, and enzymatic activity. When Ag NPs enter these fluids, proteins quickly coat their surface, changing their properties and often reducing targeting efficiency while increasing immune clearance [73]. Acidic tumor environments can promote Ag NP dissolution and aggregation, affecting Ag^+^ ion release and toxicity [74]. High salt and protein levels in blood can also cause nanoparticle clumping, reducing stability and potentially causing health risks [73]. Enzymes may degrade surface coatings, impacting circulation time and biocompatibility [75]. Oxidative conditions can change Ag NP surface chemistry, influencing toxicity [76]. To address these challenges, surface modifications like zwitterionic coatings have shown improved stability and reduced immune responses compared to PEG coatings [77]. Understanding these factors is essential for designing safe and effective Ag NP therapies in complex biological settings.

Various surface functionalization techniques, including targeting ligands, biodegradable coatings and peptide-based modifications, have been explored to optimize Ag NPs for specific therapeutic purposes and to minimize side effects [78]. For instance, Ag NP functionalization with ligands (e.g. antibodies, peptides, or aptamers) allows selectively targeting specific cells, especially in cancer therapy, thus enhancing the drug delivery efficacy and reducing off-target effects [79]. For examples, conjugation of folic acid to Ag NPs has been used to target cancer cells that overexpress folate receptors [80] and conjugation of HER2-targeting peptides for HER2-positive breast cancer therapy. Biodegradable coatings, such as poly(lactic-co-glycolic acid), chitosan and PEG, improve Ag NP stability in biological fluids and extend their circulation time, making them ideal for controlled drug delivery [18,81].

Biodegradable coatings made from materials such as polymers (e.g., PLGA, chitosan) or natural biomolecules improve the stability of Ag NPs by preventing aggregation and controlling premature Ag^+^ ion release, which reduces cytotoxicity [77]. These coatings act as protective barriers that enable a controlled release of Ag^+^ ions, minimizing acute toxicity. Additionally, they reduce immune system recognition and clearance, enhancing targeted delivery to specific tissues [82]. Moreover, biodegradable coatings promote safer excretion of Ag NPs by increasing their solubility and biodegradability, facilitating renal or hepatobiliary clearance and decreasing long-term accumulation in organs [83].

Peptide-based modifications enhance Ag NP targeting and cellular uptake through receptor-mediated endocytosis for various therapeutic applications, including cancer, cardiovascular, and neurological disorders. For example, modification with peptides derived from the HIV-1 TAT protein allows Ag NPs to cross the blood-brain barrier (BBB) for targeted delivery in neurological diseases, and RGD peptides have been used to target integrin receptors expressed on cancer cells [84]. Moreover, Ag NP surface modification with cetyltrimethylammonium bromide (CTAB), polyvinyl alcohol and hydrophilic groups improves the NP solubility and interaction with biological systems [85]. The incorporation of biomolecules (proteins or nucleic acids) on the Ag NP surface widens their potential use in drug delivery systems, diagnostics and therapeutics [86].

**Fig. 4:**
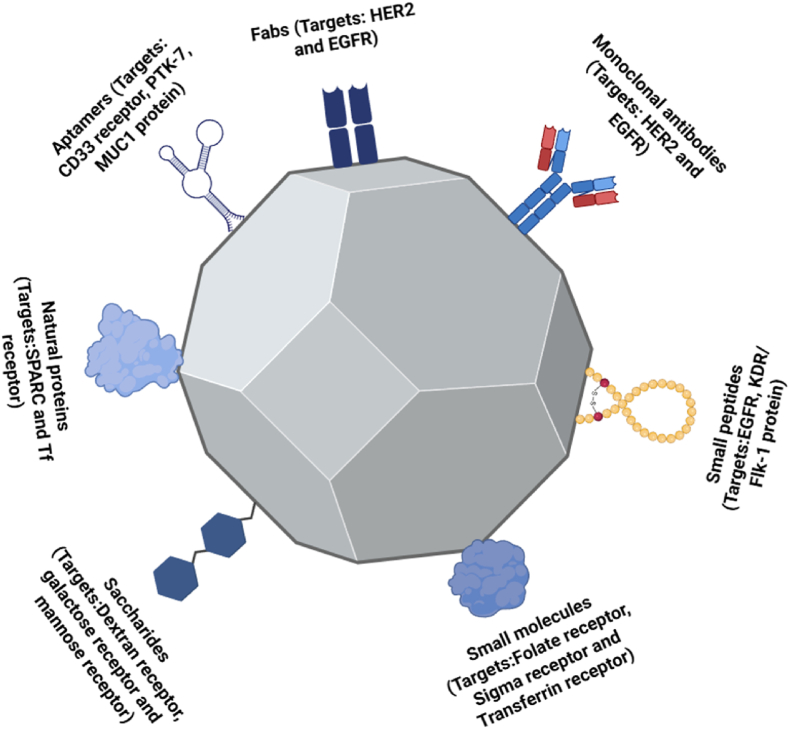
Different surface-modification strategies for Ag NP active and targeted uptake. Fab, fragment antigen binding.

Fig. 4 highlights the different strategies to enhance the Ag NP effectiveness in targeted therapies. The addition of these biomolecules (monoclonal antibodies, aptamers, carbohydrates and peptides) allows Ag NPs to selectively bind to target cells, thus improving drug bioavailability, which is particularly important in cancer therapy and immunotherapy. Table 2 provides an overview of targeting ligands and peptide-based modifications for Ag NPs, detailing their specific receptors and therapeutic applications (see Table 3).

**Table 2:** Targeting ligands and peptide-based modifications for Ag NPs with therapeutic applications.

Modification	Target	Ag NP shape and size	Target ligand/receptor	Therapeutic application	Ref
Folic acid-functionalized Ag NPs	Cancer cells (folate receptor-positive)	Spherical, 6−9 nm	Folate receptor	Drug delivery for cancer therapy	[87]
HER2-targeting peptide functionalized Ag NPs	Breast cancer cells	Spherical, ∼10–20 nm	HER2 receptor	Cancer therapy	[88]
Transferrin-functionalized Ag NPs	Cancer cells (overexpressing transferrin receptor)	Spherical, 10−15 nm	Transferrin receptor	Targeted drug delivery	[89]
TAT peptide-functionalized Ag NPs	Blood-brain barrier	Spherical, 5−10 nm		Increased drug delivery (neurodegenerative diseases)	[90]
RGD peptide-functionalized Ag NPs	Cancer cells (integrin receptors)	Spherical, 8−12 nm	Integrins	Cancer therapy	[91]
Angiopep-2 peptide-functionalized Ag NPs	Brain endothelial cells (blood-brain barrier)	Spherical, 5−15 nm	Low-density lipoprotein receptor-related protein-1 (LRP1)	Brain-targeted drug delivery	[92]

**Table 3:** Comparative Assessment of AgNP synthesis methods based on biomedical performance Criteria.

**Criteria**	**Chemical Synthesis**	**Physical Methods**	**Green Synthesis**	Ref
**Yield**	High (>90 %) under controlled laboratory conditions	Moderate to high, depends on equipment and energy input	Variable (50–85 %) depending on extract source, type, and pH	[111]
**Reproducibility**	Excellent due to standardized reagents and reaction conditions	High but dependent on precision instrumentation	Moderate to low due to variability in plant metabolites	[112]
**Cytotoxicity**	Often high due to residual chemicals such as NaBH_4_ or surfactants	Low to moderate; less residual toxicity	Low; phytochemical capping enhances biocompatibility	[113]
**Scalability**	Industrially scalable; safety and waste disposal are concerns	Limited scalability; energy-intensive and costly	Promising; eco-friendly but requires extract standardization	[100]
**Translation Potential**	High with surface modifications (e.g., PEGylation, targeting	Limited due to cost and complex instrumentation	Strong biosafety; regulatory standardization still evolving	[114]

### 2.2 Ag NP production sustainability and scalability

Scaling up the production of Ag NPs poses significant challenges, particularly to maintain consistent size, shape and quality in large-scale production. Laboratory-scale methods often struggle with reproducibility when transitioning to industrial levels. Recent advancements in scaling up Ag NP synthesis have focused on optimizing key reaction parameters, such as temperature, pH and precursor concentration, to ensure uniform particle size and shape [93]. Bioreactors for microbial-based synthesis and continuous-flow reactors for plant extract-based synthesis are promising approaches to improve uniform mixing, to better control the reaction conditions, and to reduce batch-to-batch variability. These methods allow finely tuning the Ag NP size and shape, a crucial step for various biomedical and environmental applications [94].

**Fig. 5:**
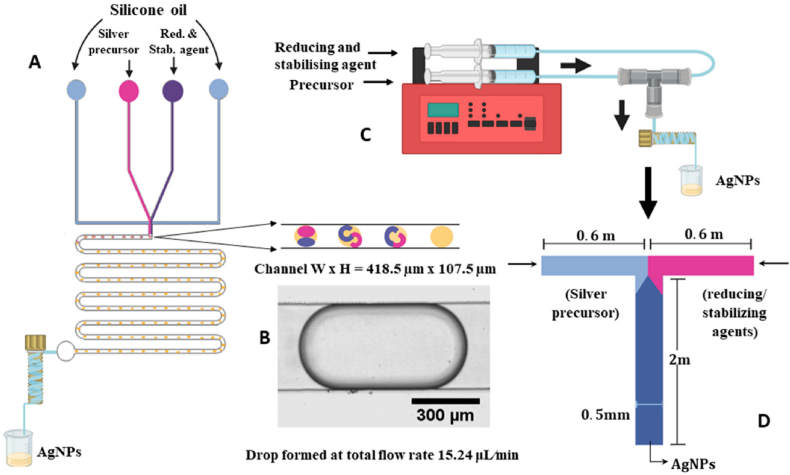
Microfluidic synthesis of Ag NPs using a flow-focusing device. (A) Schematic representation of the flow-focusing microfluidic device illustrating droplet formation within the microfluidic channels, where chemical reactions lead to Ag NP synthesis. (B) Representative image of a droplet inside the channel. (C) Experimental setup for Ag NP synthesis with the flow-focusing device. (D) Schematic representation of a T-junction microfluidic device [95]. Open Access MDPI.

To further enhance Ag NP production scalability and consistency, advanced techniques, such as continuous-flow reactors, have been proposed. These reactors can produce Ag NPs continuously once the steady state is reached, with rapid heating times (milliseconds) to better control the reaction kinetics. Fig. 5 illustrates the microfluidic synthesis of Ag NPs using a flow-focusing device [95].

The microfluidic method offers significant advantages, including better particle size control, high reproducibility and reduced reagent consumption, compared with conventional batch synthesis. Future research should focus on optimizing reaction conditions, integrating real-time monitoring techniques, and exploring novel ligand-functionalized Ag NPs for targeted therapeutic applications. Additionally, the microfluidic synthesis scalability for large-scale production remains a key research area.

Studies suggest that adjusting parameters (e.g. reducing agent concentration, stabilizer type) and processing conditions (e.g. stirring, heating and sintering) can significantly influence the Ag NP size and shape [96]. Several systematic studies have demonstrated how reaction parameters affect Ag NP characteristics. For example, increasing the concentration of reducing agents accelerates nucleation, producing smaller and more uniform nanoparticles, while lower concentrations favor larger particle growth [97]. The type and amount of stabilizers, such as polyvinylpyrrolidone (PVP), citrate, or chitosan, play key roles by providing functional groups (C=O, -COOH, -OH, and -NH_2_), which enhance colloidal stability and surface charge, prevent aggregation, and improve biocompatibility [98]. Additionally, pH and temperature significantly affect the reduction rate and crystallinity of Ag NPs, directly influencing their nucleation and growth dynamics. Higher temperatures typically increase the reduction rate, leading to faster nucleation and smaller particles, while pH can alter the ionization state of stabilizing agents’ functional groups, impacting their binding affinity to the nanoparticle surface and thus their stabilization efficiency [99]. These factors collectively determine the final particle size distribution, morphology, and crystallinity, which are critical for the functional performance and stability of Ag NPs in applications.

Additionally, scaling up Ag NP synthesis requires careful management of waste and by-products generated during the process. Common reagents such as silver salts, reducing agents (e.g., citrate, sodium borohydride, hydrazine), and stabilizers (e.g., PVP, PEG) can produce hazardous residues that must be properly treated to prevent environmental contamination [100]. To enhance sustainability, current efforts focus on using renewable raw materials like plant extracts or biodegradable polymers as reducing and capping agents, which reduce reliance on toxic chemicals and lower waste toxicity. Recycling and reusing by-products, including unreacted Ag^+^ ions, further minimize environmental impact and improve cost efficiency [101]. Furthermore, life-cycle assessments are increasingly employed to evaluate and reduce the environmental footprint of Ag NP synthesis methods, guiding the development of more sustainable production practices [102].

### 2.3 Evaluation of Ag NP synthesis techniques for biomedical applications

Chemical-based synthesis methods of Ag NPs, such as the reduction of silver salts using sodium borohydride or citrate, are widely adopted due to their high reaction efficiency, reproducibility, and scalability. These techniques typically yield Ag NPs in the range of 80–95 %, with excellent control over particle size, morphology, and surface properties. As a result, chemically synthesized Ag NPs often exhibit high uniformity, stability, and tunability, making them particularly suitable for biomedical applications requiring precision, such as targeted drug delivery, biosensing, and diagnostic imaging. However, a key limitation lies in the use of toxic chemical precursors and stabilizing agents, which necessitate rigorous post-synthesis purification to avoid residual toxicity [103]. These impurities, if not adequately removed, can lead to cytotoxic effects and limit the safe use of chemically synthesized Ag NPs in clinical settings [104].

Biological or green synthesis methods involve the use of plant extracts, bacteria, fungi, or other natural reducing agents to produce silver nanoparticles in an eco-friendly and safe manner. Mycogenic synthesis primarily involves fungi such as *Fusarium*, *Aspergillus*, *Penicillium*, *Trichoderma*, *Cladosporium*, *Rhizopus*, and *Ganoderma*, which secrete extracellular enzymes like nitrate reductase and various metabolites that reduce silver ions (Ag^+^) to metallic silver (Ag^0^) and stabilize the nanoparticles. Similarly, bacterial species including *Bacillus*, *Pseudomonas*, *Escherichia coli*, *Streptomyces*, and *Lactobacillus* produce enzymes and proteins capable of reducing and capping silver nanoparticles. Other microbes, such as algae and actinomycetes, also contribute to green synthesis through their bioactive compounds. These biological methods are gaining significant attention due to their sustainability, cost-effectiveness, and reduced environmental impact compared to traditional chemical synthesis [104,105].

Biological synthesis approaches eliminate the need for hazardous chemicals and often result in nanoparticles capped with naturally occurring biomolecules such as flavonoids, terpenoids, and polysaccharides, which can enhance cellular compatibility and reduce toxicity. Despite these advantages, green synthesis methods generally produce lower and more variable yields, typically ranging from 30 % to 80 %, due to slower reaction kinetics and the complex, often poorly defined nature of biological reducing agents [105]. Additionally, these methods are more susceptible to batch-to-batch variability, as the composition of biological extracts can fluctuate with factors such as season, geographic location, and extraction conditions. This inconsistency can affect critical nanoparticle parameters including size, surface charge, and functional group composition which are essential for reproducibility and therapeutic efficacy [106].

Considering their potential for clinical use, chemically synthesized Ag NPs remain dominant in preclinical research due to the availability of standardized protocols and the ability to finely tune physicochemical properties for specific applications [107]. However, their clinical translation is frequently hindered by concerns related to toxicity, bioaccumulation, and environmental impact. In contrast, green-synthesized Ag NPs offer enhanced biosafety and ecological acceptability, aligning well with modern sustainability goals [108]. Nevertheless, for these biogenic Ag NPs to be adopted in mainstream biomedical applications, standardization of synthesis protocols, improved scalability, and compliance with Good Manufacturing Practice (GMP) are crucial [109,110]. Overall, the choice between chemical and green synthesis should consider not only yield and scalability but also biocompatibility, regulatory feasibility, and the intended biomedical application.

## 3 Ag NPs as smart therapeutic agents

**Fig. 6:**
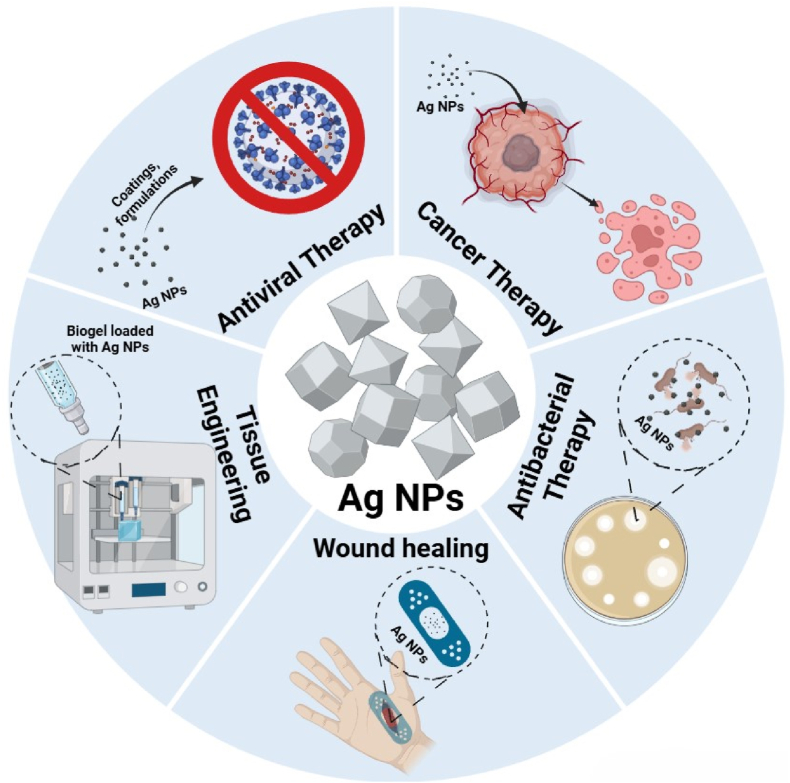
Ag NPs as smart therapeutic agents for targeted drug delivery, controlled release, and enhanced efficacy in antimicrobial, antiviral, and anticancer therapies.

Ag NPs are considered smart therapeutic agents because they can enhance drug delivery through precise targeting, controlled release and responsiveness to environmental stimuli [71]. Therefore, Ag NPs are a preferred choice in modern therapeutic strategies, offering innovative solutions for cancer treatment, infection control, tissue repair, and more [115]. Their unique physicochemical properties are strongly size-dependent [116]. Typically, Ag NPs range from **1 to 50 nm**, exhibiting distinct optical and catalytic behaviors such as **localized surface plasmon resonance (LSPR).** When reduced to **nanoclusters** (approximately 1–2 nm), these particles contain only limited numbers of atoms and display **quantum behaviors** resembling molecular systems, without classical plasmonic activity [117]. Further down the scale, **angstrom-scale particles** (less than 1 nm or under 10 Å) exhibit properties that closely to clusters of individual atoms, where quantum effects dominate entirely. At the smallest scale, **individual ions** represent single atoms or molecules and lack any nanoparticle-specific characteristics. This size-dependent tunability significantly influences Ag NPs' reactivity, biological interactions, and therapeutic efficacy [118]. As shown in Fig. 6, Ag NPs can be engineered for targeted delivery, controlled release, and enhanced therapeutic effects in antimicrobial, anticancer, and tissue engineering applications.

### 3.1 Responsive drug delivery systems

Stimuli-responsive drug delivery systems have revolutionized treatments by allowing the precise, targeted drug release in response to environmental triggers (e.g. pH, temperature, enzymes). These advanced systems enhance therapeutic efficacy and minimize side effects, compared with traditional treatments [119]. For instance, pH-sensitive systems release drugs in the acidic tumor microenvironment and temperature-responsive systems are activated in injured/tumor tissues where the local temperature is increased. Recent innovations have further refined drug delivery system precision, optimizing drug bioavailability and site-specific treatments [120]. Among different metal and metal oxide NPs used in RDDS (Au, ZnO, CuO, Fe_3_O_4_), Ag NPs stand out for their strong antimicrobial activity and ease of surface modification [121]. Unlike Au NPs, which offer light-triggered release with superior biocompatibility, Ag NPs are more cost-effective and easier to synthesize via green routes [122].

Ag NP-based drug delivery systems or antibodies enhances their interaction with biological targets, improving drug delivery efficacy [115]. Additionally, their antibacterial properties are useful for infection management, and their biocompatibility ensures their safe use in various therapeutic contexts. Ag NP size (from 5 nm to 100 nm) can influence their cellular uptake, drug release and antibacterial performance. Smaller NPs (5 nm) penetrate plasma membranes quickly and distribute uniformly in the cytoplasm, whereas larger NPs (100 nm) show different uptake efficiency and distribution [123]. Ag NPs of different sizes exhibit different cellular uptake efficiencies [120]. For example, 100 nm Ag NPs have the highest uptake efficiency in B16 cells, whereas 5 nm Ag NPs cross the plasma membrane faster and distribute more uniformly in the cytoplasm and nucleus. Similarly, the shape of Ag NPs influences their antibacterial performance: nanocubes (∼50 nm) show the highest activity, followed by nanospheres (∼20 nm) and nanowires (∼100 nm) [124]. Goyal, Kaur, Tewari and Kumar [125] observed that triangular (prismatic) and hexagonal NPs (∼30–80 nm) with sharper edges exhibit superior antibacterial activity due to their enhanced interaction with bacterial membranes Seyedpour, Arabi Shamsabadi, Khoshhal Salestan, Dadashi Firouzjaei, Sharifian Gh, Rahimpour, Akbari Afkhami, Shirzad Kebria, Elliott, Tiraferri, Sangermano, Esfahani and Soroush [126] demonstrated that supramolecular structures of silver-based metal-azolate frameworks further enhance antibacterial efficacy. Functionalization with biomolecules (e.g. chitosan, folic acid, peptides) improves receptor-mediated uptake and selective accumulation at target sites, thereby minimizing systemic toxicity. For example, PEGylated Ag NPs (∼10–50 nm) exhibit prolonged circulation time and reduced aggregation, enhancing their stability in physiological environments. Chitosan-coated Ag NPs (∼15–40 nm) show improved antibacterial activity and biocompatibility due to their positive surface charge, which facilitates electrostatic interactions with bacterial cell membranes. Such modifications are essential for designing Ag NP-based drug delivery systems that maximize therapeutic efficacy and minimize off-target effects [127].

**Fig. 7:**
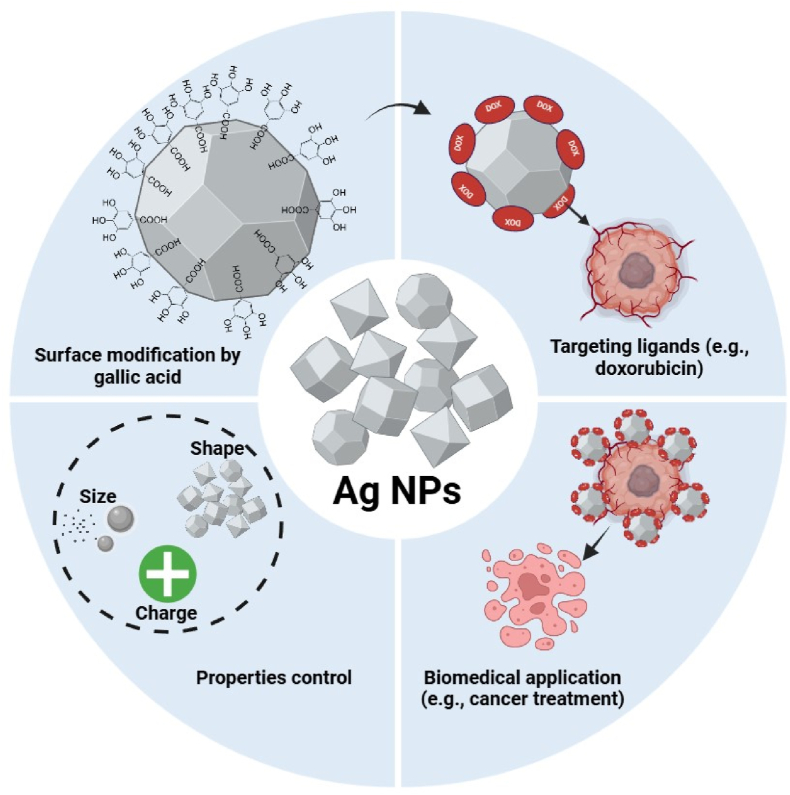
Schematic presentation of gallic acid-coated Ag NPs for drug delivery, highlighting their stability, drug-loading capacity and controlled release mechanisms for targeted therapies.

Fig. 7 shows the potential of gallic acid-coated Ag NPs as drug nanocarriers due to their enhanced stability, controlled drug release and efficient targeting [128]. Coating with gallic acid improves the Ag NP biocompatibility and enhances their drug-loading capacity, making them ideal for controlled and targeted drug delivery. These NPs can be used to deliver therapeutic agents effectively, while minimizing off-target effects. Table 4 provides a detailed summary of Ag NP stimuli (pH, light, temperature, enzymes), the mechanisms through which they release drugs in response to these stimuli, and their advantages (enhanced specificity, reduced toxicity, controlled and sustained drug release) for targeted treatments.

**Table 4:** Examples of the use of Ag NPs in responsive drug delivery, stimuli, mechanisms, and advantages.

Disease	Active ingredient	Stimulus	Delivery mechanism	Ag NP shape and surface functionalization	Advantages	Ref
Inflammatory bowel disease	Mebeverine precursor	pH (mimicking colon conditions)	Controlled release in inflamed colon	Spherical Ag NPs (10–17 nm); galactose	Targeted delivery, reduced side effects, improved efficacy	[129]
Cancer therapy	Doxorubicin, cisplatin, epirubicin	Magnetic field, temperature, pH, secreted phospholipase A2	Stimulus-responsive nanocarriers (liposomes, micelles)	Spherical and triangular Ag NPs (20–80 nm); graphene oxide	Enhanced tumor targeting, controlled release, reduced cardiotoxicity	[130]
Tumor-specific drugs	Chemotherapy agents	Endogenous (pH, enzymes) or exogenous (light, magnetic field)	Payload release at the tumor site	–	Enhanced therapeutic effect, reduced resistance	[131]
Bacterial infections	Ag NPs	Light (UV)	Antimicrobial ion release via ROS generation	Spherical Ag NPs (5 nm); amines	Higher antimicrobial activity, minimal human cell toxicity	[132]
Pseudomonas infections	Azlocillin	Combination therapy with Ag NPs	Increased antibacterial activity, reduced minimum inhibitory concentration (MIC)	Spherical Ag NPs (50–60 nm); glutathione	Enhanced potency against *P. aeruginosa*	[133]
Multi-antibiotic therapy	Chloramphenicol, kanamycin, biapenem, aztreonam, ampicillin	Combination therapy with Ag NPs	Synergistic MIC reduction	Spherical Ag NPs (15–35 nm); polyvinylpyrrolidone (PVP)	Overcoming bacterial resistance	[134]
*E. coli* and *S. aureus* infections	Vancomycin, amikacin	Functionalized Ag NPs	Synergistic effects, overcoming resistance	Spherical Ag NPs (5–35 nm); PVP	Effective against resistant strains	[135]
Antibiotic-resistant infections	Vancomycin, ampicillin, penicillin	Combination with Ag NPs	Enhanced antibacterial activity	Spherical Ag NPs (23–33 nm); polymers	Effective against *S. aureus*, *E. coli*, *K. pneumoniae*	[136]
Ampicillin-resistant strains	Ampicillin	Functionalized Ag NPs	Reduced colony-forming unit value in resistant strains	Spherical Ag NPs (4 nm); ampicillin	Effective against susceptible and resistant strains	[137]
Skin infections	Ag NPs	Temperature	Controlled ion release via a temperature-sensitive hydrogel	Spherical Ag NPs (5–20 nm); polycaprolactone	Faster wound healing	[132]
Chronic infections	Ag NPs	Enzyme-induced	Localized release via an enzyme-degradable matrix	Spherical Ag NPs (100–110 nm); CTAB-modified porous silica	Controlled release at the infection site	[132]
Respiratory infections	Ag NPs	Magnetic field	Directed release at the infection site	Spherical Ag NPs (3–10 nm); PVP	Localized delivery, reduced side effects	[132]
Multi-drug resistant infections	Erythromycin, ampicillin, chloramphenicol, cephalothin, clindamycin, tetracycline, gentamycin, amoxicillin, ciprofloxacin, cefpodoxime, cefuroxime	Combination with Ag NPs	Synergistic action	Spherical Ag NPs (72 nm); glycyrrhizin coating	Turning resistant strains into strains susceptible to treatment	[138]

### 3.2 Theranostics

Ag NPs ability to improve imaging techniques, such as CT and surface-enhanced Raman scattering (SERS), enhances tumor detection and allows more precise targeted treatments, including photothermal therapy and precision drug delivery. In neurotheranostics, their ability to cross the BBB is crucial for targeted imaging and treatment of neurological disorders, such as Alzheimer's disease. Furthermore, in infectious disease management, Ag NPs act as biosensors for pathogen detection and simultaneously deliver antimicrobial agents. The versatility of Ag NPs in these applications highlights their potential to revolutionize the future of diagnostics and therapeutics, offering an integrated approach to patient care [139].

The size of Ag NPs plays a crucial role in their theranostic applications because smaller particles interact more effectively with cells and tissues [140]. Smaller Ag NPs (<10 nm) exhibit enhanced cellular uptake and stronger anticancer effects due to their high surface area-to-volume ratio and efficient ROS generation. Spherical Ag NPs offer excellent biocompatibility and stability, while triangular and rod-shaped Ag NPs have superior plasmonic properties, ideal for photothermal therapy, imaging and LSPR biosensing. Triangular nanoplate arrays are suitable for biosensing applications. Moreover, flat-topped Ag NPs display stronger antibacterial activity than spherical Ag NPs. Ag nanorods and flower-shaped Ag NPs, synthesized by chemical reduction, have tunable LSPR properties for theranostic applications [92]. These structural variations offer the opportunity to control Ag NP functionality in order to optimize their potential for targeted drug delivery, imaging, and biosensing in advanced medical applications [141].

Beyond monometallic Ag NPs, recent advances emphasize the development of multifunctional hybrid platforms. Core–shell Au@Ag NPs have been widely studied for their synergistic plasmonic effects, combining the superior photothermal efficiency of gold with the strong optical tunability and biosensing capabilities of silver. These structures offer integrated dual functionality for simultaneous imaging and therapeutic delivery (e.g., photothermal ablation of tumors with real-time imaging guidance) [142]. Adding magnetic properties to Ag NPs enables simultaneous imaging and therapy, offering unique advantages for precision oncology and other medical fields. For instance, Fe_3_O_4_-Ag quantum dot nanohybrids demonstrate enhanced MRI contrast and fluorescence intensity due to the optimized energy transfer between magnetic and fluorescent components [143]. However, challenges remain in optimizing the interactions between these properties to prevent potential interference or functional attenuation, highlighting the need for rigorous experimental validation. Techniques such as fluorescence spectroscopy, superconducting quantum interference device (SQUID) magnetometry, and *in vitro* studies are essential for ensuring that the combined functionalities perform optimally in complex biological environments [144].

Different composite Ag NPs have recently gained significant attention due to their enhanced physicochemical properties and multifunctionality, making them ideal for biomedical applications. Nowadays, circulating tumor cells (CTCs), which are rare cancer cells released from primary tumors into the bloodstream, serve as crucial liquid biomarkers for cancer diagnosis and prognosis [[145], [146], [147]]. To improve the detection of CTCs, Pang et al. developed a magnetically assisted SERS biosensor using a composite of anti-ASGPR antibody-functionalized Fe_3_O_4_@Ag magnetic nanoparticles combined with anti-GPC3 antibody-coated Au@Ag@DTNB nanorods. This dual-component system exhibited outstanding sensitivity, achieving a detection limit of just 1 cell/mL in blood samples from hepatocellular carcinoma patients. The synergistic effect of magnetic separation and SERS enhancement, along with dual antibody specificity, highlights the importance of composite Ag NPs in advancing highly sensitive and selective cancer diagnostics today [148].

In parallel, Chang et al. recently introduced a time-efficient method to synthesize Au nanobipyramid@Ag (Au NBP@Ag) core–shell nanorods exhibiting strong SERS activity and monodispersity. These nanoprobes, functionalized with the Raman reporter MBA and folic acid-conjugated bovine serum albumin (rBSA-FA), enabled rapid and reproducible identification of live cancer cells (MGC-803) via high-resolution SERS mapping. Their high specificity was further confirmed against non-cancerous cells (A549), underscoring their clinical potential for early cancer detection [149]. Additionally, Ag NPs are being engineered and used into advanced delivery systems incorporating immunotherapy agents such as anti-PD-L1 antibodies and CRISPR-Cas9 gene-editing tools. These hybrid nanoplatforms facilitate simultaneous tumor imaging, immune modulation, and gene editing, positioning Ag-based theranostics at the forefront of personalized cancer nanomedicine [150].

**Fig. 8:**
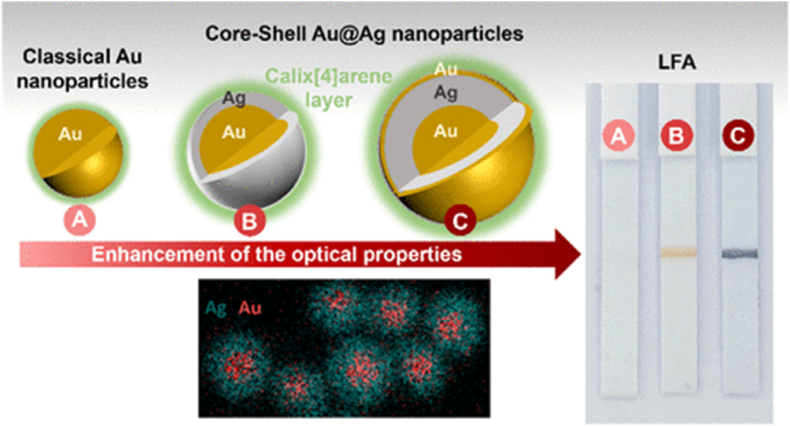
Tailored ultra-stable core–shell Au@Ag NPs to improve colorimetric detection in lateral flow assays (LFA) [152]. Reproduced with permission from ACS.

Au@Ag NPs can be synthesized using various methods, e.g. core–shell growth, galvanic replacement, and seed-mediated growth techniques [151]. In the core–shell growth method, a thin Ag shell is deposited onto preformed Au NPs by reducing Ag^+^ ions in the presence of stabilizing or capping agents, allowing for precise control over shell thickness. These core–shell Au@Ag NPs have demonstrated significant potential in improving colorimetric signal amplification in lateral flow assays (LFAs). As illustrated in Fig. 8, Au@Ag NPs enhance colorimetric detection sensitivity by leveraging the synergistic plasmonic properties of both metals [152]. The gold core offers excellent optical stability and biocompatibility, while the silver shell significantly amplifies the SPR, resulting in brighter signals and greater contrast. This dual-metal architecture boosts the localized electromagnetic field, thereby enhancing light scattering and absorption. Such plasmonic enhancement enables lower detection limits, making it possible to visually identify even trace concentrations of target analytes. This is especially valuable in point-of-care diagnostics, where rapid and reliable visual results are essential. Moreover, the engineered surface of Au@Ag NPs can be functionalized with specific antibodies or aptamers to ensure high specificity and minimal background interference, further improving the assay's accuracy and reproducibility [152].

**Fig. 9:**
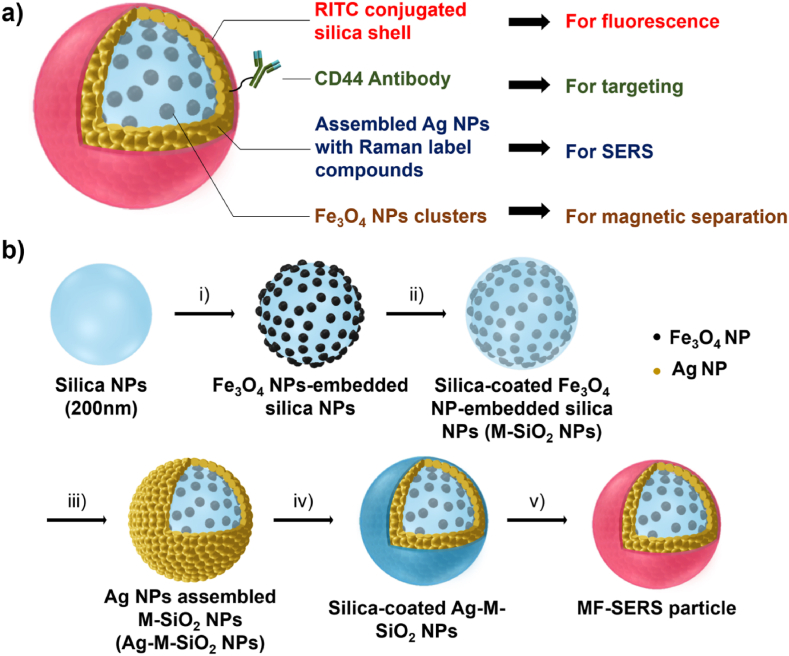
Assembly of NPs with a fluorescent Ag-Fe_3_O_4_-silica shell layer for tri-functional magnetic-fluorescence-SERS (MF-SERS) probes and their applications. (A) Structure of a MF-SERS particle. (B) Synthesis steps of MF-SERS particles: (i) introduction of magnetic (M) Fe_3_O_4_ NPs on the silica NP surface, (ii) silica coating, (iii) Ag NP addition, (iv) silica coating, and (v) addition of the fluorescent shell layer (rhodamine B isothiocyanate, RITC) [156]. Open Access, Springer Nature.

Rapid advancements in nanomedicine have led to the development of multifunctional hybrid NPs that integrate various properties into a single platform, offering enhanced diagnostic and therapeutic capabilities [153]. The development of Fe_3_O_4_/Ag/SiO_2_ NPs marks a significant advancement in nanomedicine, by integrating magnetic, plasmonic and fluorescent functionalities into a single theranostic platform [154]. The Ag shell enhances SERS and LSPR, improving biosensing sensitivity and photothermal therapy. The SiO_2_ layer stabilizes the NPs and provides a biocompatible surface for functionalization with targeting ligands, improving the cell specificity. Spherical Fe_3_O_4_/Ag/SiO_2_ NPs offer excellent stability and homogeneity for bioimaging and controlled drug release, while anisotropic structures (nanorods or nanoplates) enhance the optical properties for deep-tissue applications. The SiO_2_ layer fluorescence can be tuned for real-time imaging and biodistribution monitoring. These hybrid NPs allow simultaneously magnetic resonance imaging contrast enhancement, biomarker detection via SERS and photothermal therapy, positioning them as a powerful tool for precision medicine [155]. Fig. 9 shows the versatility of these NPs.

## 4 Ag NPs in regenerative medicine and tissue engineering

**Fig. 10:**
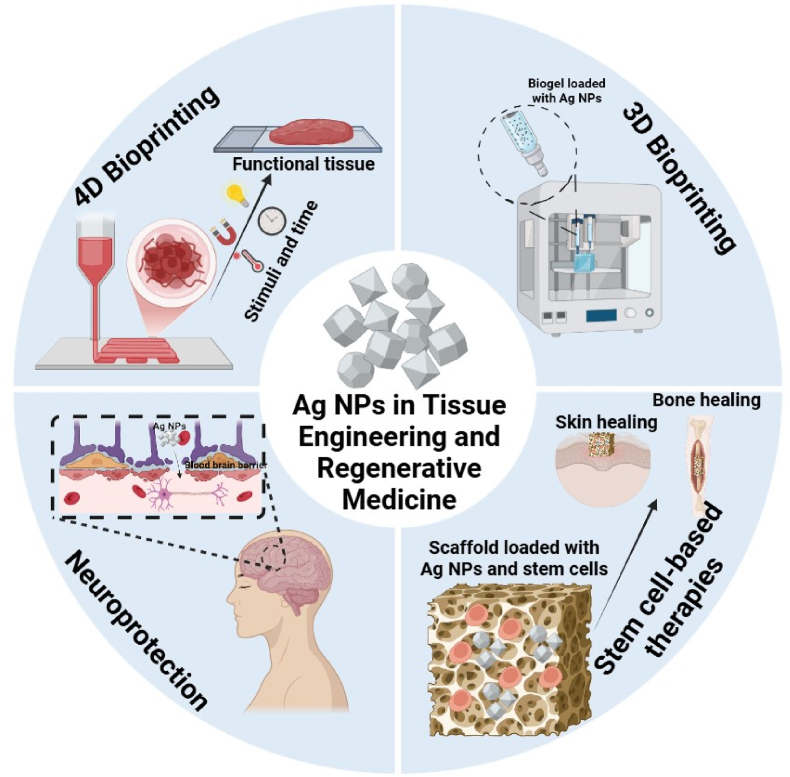
Applications of Ag NPs in regenerative medicine and tissue engineering.

Ag NPs have emerged as key tools in regenerative medicine and tissue engineering. The effectiveness of Ag NPs in these applications is influenced by their size, shape and surface functionalities [157]. These physicochemical properties dictate their interactions with biological systems, influencing their biocompatibility, antimicrobial efficacy and cellular interactions [21]. For instance, variations in Ag NP size and shape can affect their dissolution rate, thereby influencing their antibacterial activity. Moreover, surface functionalization plays a crucial role in determining the cellular uptake and overall biological response to these NPs. By tailoring these parameters, Ag NPs can be optimized as indispensable tools for developing innovative therapies in regenerative medicine and tissue engineering [158]. Compared to gold Au NPs, which are often used for their inertness and imaging capabilities, Ag NPs actively modulate cellular responses and prevent microbial contamination [159]. While ZnO and CuO NPs contribute to osteogenesis and angiogenesis, Ag NPs offer superior antimicrobial protection but may present cytotoxicity at higher concentrations, requiring careful dose optimization in tissue scaffolds [160,161]. Fig. 10 displays the diverse applications of Ag NPs in neuroprotection, stem cell-based therapies, and 3D and 4D bioprinting.

### 4.1 Ag NP neuroprotective and neurodegenerative properties

Ag NPs are promising candidates for treating neurodegenerative diseases such as Alzheimer's and Parkinson's due to their ability to cross the blood–brain barrier (BBB) and their antioxidant and anti-inflammatory properties [162]. The anti-inflammatory effect of a topical nanocrystalline silver cream was tested in a mouse model of allergic contact dermatitis. When researchers compared nano-crystalline silver to steroids and immunosuppressants, they discovered that it significantly reduced erythema in a concentration-dependent way. It reduces TNF-α and IL-12 levels and causes inflammatory cells to die [163]. Their physicochemical characteristics: particularly size, shape, and surface functionalization, significantly influence BBB permeability and therapeutic efficacy. Studies have shown that Ag NPs can cross the BBB via receptor-mediated and adsorptive-mediated endocytosis, processes that can be enhanced through surface modification with targeting ligands like TAT or angiopep-2 peptides [164]. Smaller particles (<20 nm) exhibit higher permeability and more uniform distribution in brain tissue, while spherical NPs are generally more efficient at crossing the BBB than anisotropic shapes due to their favorable interaction with endocytic pathways. Functional coatings also improve targeting and reduce cytotoxicity [165] For instance, peptide-coated spherical Ag NPs (∼30 nm) have demonstrated enhanced delivery to neural tissues, supporting their potential for future neurological therapies [85].

### 4.2 Stem cell-based therapy

In stem cell-based therapies, Ag NPs can significantly influence the tissue regeneration outcomes. Their physicochemical properties (size, shape, and surface functionality) play pivotal roles in modulating the stem cell behavior [166]. The higher cellular uptake rates of smaller Ag NPs (from 20 to 70 nm) promotes the differentiation of mesenchymal stem cells into osteoblasts, thus facilitating bone regeneration [167]. Ag NP shape also affects their internalization into stem cells. Nanospheres are internalized more efficiently than nanorods or quasi-ellipsoid NPs of similar size, influencing stem cell differentiation [168]. Surface modifications, such as coating with biocompatible polymers or peptides, can further enhance stem cell differentiation and reduce cytotoxic effects. Additionally, incorporating Ag NPs into scaffolds made of biomaterials provides structural support and improves stem cell attachment, proliferation and differentiation, promoting tissue regeneration. Tailoring Ag NP physicochemical properties is essential for maximizing the regenerative potential of stem cell-based therapies [158].

### 4.3 3D and 4D bioprinting

Ag NP antibacterial and regenerative potential are exploited to significantly advance 3D and 4D bioprinting technologies for tissue engineering. In 3D bioprinting, Ag NPs enhance cell viability by promoting adhesion, proliferation, and differentiation, leading to functional tissues (bone, cartilage, and skin) [169]. Their antimicrobial properties prevent bacterial contamination during fabrication and after implantation, reducing the infection risk. For instance, spherical Ag NPs (∼70 nm) coated with polydopamine significantly limit *S. aureus* colony formation, enhancing the antimicrobial properties of printed scaffolds. Polydopamine-coated Ag NPs also improve osteoblast-like cell proliferation, suggesting their potential in bone tissue engineering and biomedical implants [170]. Ag NPs also reinforce bioinks and scaffolds, improving the mechanical strength for load-bearing applications [171]. In 4D bioprinting, where dynamic structures responsive to external stimuli are created, Ag NPs enable the constructs to change shape or function based on environmental cues, enhancing their adaptability and functionality [172]. This is particularly beneficial for smart wound dressings and self-healing tissues. However, several challenges, including the Ag NP potential cytotoxicity and non-uniform distribution, must be overcome. Ongoing research is focused on optimizing Ag NP formulations, exploring advanced surface modifications, and developing hybrid bioinks with tailored properties to maximize the therapeutic efficacy of bioprinted tissues and organs [166]. Table 5 lists some of the challenges of Ag NPs in regenerative medicine and tissue engineering.

**Table 5:** Ag NP roles, applications, and challenges in regenerative medicine and tissue engineering.

Applications	Ag NP role	Ag NP size/shape	Ag NP surface functionalization	Examples	Challenges	Ref
**Neuroprotection & neurodegeneration**	Crossing the BBB, reducing oxidative stress, promoting axonal growth	Spherical, 19 ± 8 nm	Capped with biomolecules from black peel pomegranate extract	Amyloid-beta aggregation inhibition in Alzheimer's disease, nerve regeneration promotion	Cytotoxicity at high concentrations, potential persistence in the central nervous system	[173]
**Stem cell-based therapy**	Providing biochemical cues for stem cell differentiation, accelerating tissue healing, enhancing scaffold performance	Spherical, ∼30 nm	Embedded in a polymeric scaffold with collagen, chondroitin sulfate, and fibronectin	Mesenchymal stem cell osteogenesis and cartilage regeneration promotion	Controlled release of Ag^+^ ions, managing long-term effects on stem cells.	[174]
**3D Bioprinting**	Improving cell viability, providing antimicrobial protection, strengthening mechanical properties	Spherical, 10–30 nm	Functionalized with a silica-based bioactive glass matrix	Fabrication of skin and bone constructs and antibacterial scaffolds; tensile strength increase	Uniform NP distribution, potential cytotoxicity during printing	[175]
**4D Bioprinting**	Adding stimulus-responsive functionalities, sustained antibacterial activity, supporting self-healing tissues	Not specified	Employed in handheld bioprinters for *in situ* wound healing	Smart wound dressings, dynamic cartilage implants, soft robotics for drug delivery	NP aggregation in stimuli-responsive materials, optimizing biocompatibility	[176]

## 5 Wound healing and antimicrobial resistance with Ag NPs

Effective wound healing critically depends on controlling microbial infections, as bacterial colonization can delay tissue repair and lead to severe complications [176]. Antimicrobial agents are therefore important components of wound management to prevent infection and promote faster regeneration. Among these, Ag NPs have gained significant attention due to their broad-spectrum antimicrobial activity, ability to disrupt bacterial membranes, and low likelihood of inducing resistance [132].

Ag NPs exhibit strong antimicrobial activity through multiple mechanisms, including disruption of bacterial membranes, generation of ROS, and release of Ag^+^ ions. Ag NPs constitute a highly promising platform for the development of advanced antimicrobial systems. Their antibacterial efficacy is not only attributed to their direct interaction with microbial surfaces but is also largely governed by the release of Ag^+^ ions via oxidative dissolution [177]. This process is driven primarily by the presence of oxygen and is highly dependent on the surface chemistry of the nanoparticles. Recent studies confirm that the dissolution of Ag^0^ into Ag ^+^ plays a central role in microbial toxicity, as Ag^+^ interacts with thiol-containing proteins in bacterial membranes, disrupts enzymatic function, and generates oxidative stress. The release kinetics of Ag^+^ ions are influenced by factors such as nanoparticle size, shape, aggregation behavior, and surface functionalization [178]. Notably, halide and sulfide ions in the biological environment can bind to Ag^+^, forming insoluble salts (e.g., AgCl, Ag_2_S), thereby reducing its bioavailability and antimicrobial potency. Therefore, controlling surface passivation, functional group coating, and colloidal stability is critical for optimizing therapeutic efficacy [179].

**Fig. 11:**
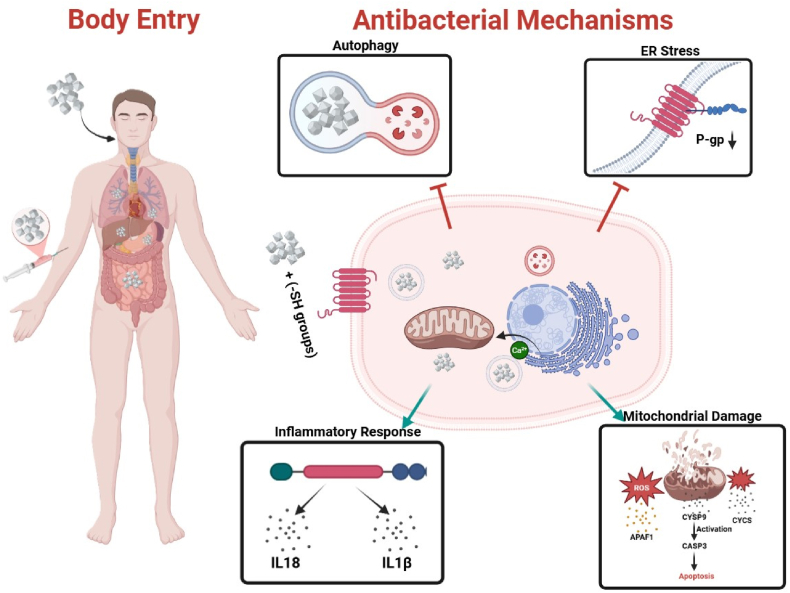
Proposed antibacterial mechanisms of Ag NPs, including ROS generation, mitochondrial and ER stress, and apoptosis via CYCS, CASP3, and APAF1. AgNPs also influence inflammatory mediators (IL18, IL1β), drug efflux (P-gp), and metabolic enzymes (CYSP9).

Understanding the mechanism of action involves two interconnected steps: (1) the behavior of Ag NPs in complex biological or environmental matrices, where aggregation, dissolution, and surface reactions may occur, and (2) the cellular interactions resulting from Ag^+^ release, ROS generation, and membrane-targeted effects. This dual-phase understanding supports the rational design of Ag NP-based systems with enhanced selectivity and sustained antimicrobial action. Recent direct observations have underscored that even trace levels of oxidative Ag^+^ release are sufficient to trigger bactericidal effects, provided the nanoparticle surface is appropriately engineered [180]. The antibacterial mechanisms associated with Ag NPs are illustrated in Fig. 11.

They interact electrostatically with negatively charged sits in the bacterial cell walls, leading to structural damage [181]. The Ag NPs also catalyze the formation of ROS (O_2_^−^, •OH, H_2_O_2_) causing damage to bacterial DNA, proteins, and lipids, and finally lead to cell death. The kinetics of the reactions may vary depending on particle size, shape, and surface functionalization. For instance, smaller Ag NPs (<10 nm) may exhibit enhanced antibacterial activity due to their higher ability for membrane penetration and ROS production [182,183]. Shape also influences antimicrobial activity, for example, the triangular Ag NPs (∼25 nm) displays significantly higher activity than spherical or rod-shaped counterparts. This is attributed to their sharp edges and high surface energy that promote stronger interaction with bacterial membranes [157]. Surface functionalization further enhances antimicrobial activity of Ag NPs. For example, Ag NPs modified with cationic ligands like polyethyleneimine (PEI) or quaternary ammonium compounds improve bacterial membrane penetration and disrupt biofilms [184]. Plant-derived phenolic and flavonoid coatings stabilize Ag NPs and promote ROS-mediated bacterial killing [185]. Moreover, conjugation of Ag NPs with antibiotics (e.g. ceftriaxone or ampicillin) yields synergistic effects, reducing the minimum inhibitory concentration (MIC) and enhancing their action against resistant strains [186].

**Fig. 12:**
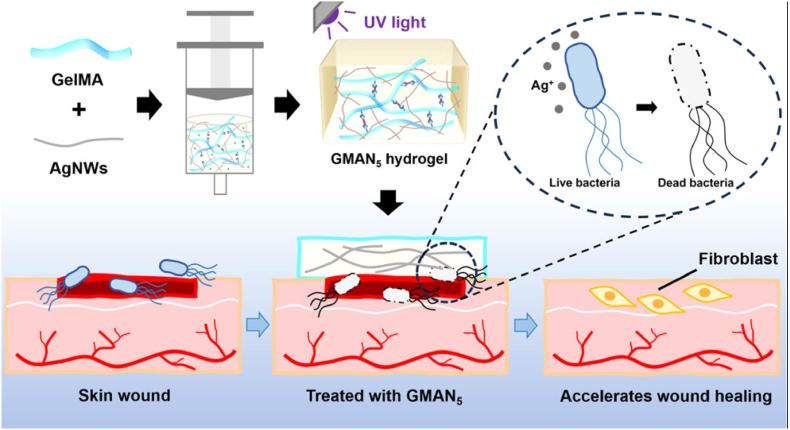
Wound healing is accelerated by silver nanowire (AgNWs)–doped gelatin methacryloyl (GelMA) dressings. These dressings combine AgNW antimicrobial activity and GelMA biocompatibility and mechanical support to promote faster wound closure and tissue regeneration. Ultraviolet (UV) crosslinking increases their stability and adhesion to the wound site [188]. Reproduced with permission from ACS.

As shown in Fig. 12, silver nanowires embedded in UV-crosslinked gelatine methacrylate dressings enhance wound healing by providing both antimicrobial activity and structural support. The antibacterial efficacy of Ag NPs is closely linked to their physicochemical properties, and fine-tuning these parameters is essential for maximizing their action against conventional and multidrug-resistant pathogens [187]. Ag NPs enhance wound healing through a multifaceted mechanism: they eliminate pathogens by disrupting bacterial membranes and generating reactive oxygen species (ROS), while simultaneously modulating the immune response by downregulating pro-inflammatory cytokines such as TNF-α, IL-6, and IL-1β. This balanced immunomodulation prevents excessive inflammation and promotes tissue repair. Furthermore, AgNPs stimulate fibroblast proliferation, collagen production, and angiogenesis; leading to faster and cleaner wound closure with minimal scarring [187].

## 6 Ag NPs in gene therapy

Due to their unique physicochemical properties (small size, large surface area, and tunable surface chemistry), Ag NPs have a transformative role in gene therapy, enabling significant advances in cancer therapy and plant genome engineering. Gene therapy typically relies on gene silencing, gene replacement, and genome editing in which genetic material is modified using mRNAs, plasmids and nucleases, such as zinc-finger nucleases, TALE nucleases and the CRISPR/Cas-9 system [189].

Functionalized Ag NPs have been widely explored for increasing gene therapy delivery efficiency. One approach involves the use of PEI-capped Ag NPs and an antiviral small interfering RNA (siRNA) small interfering RNA to inhibit the EV71 virus, which causes hand-foot-and-mouth disease. Such Ag@PEI@siRNA nanocomposites demonstrated minimal cytotoxicity and blocked viral replication via ROS-mediated signaling, making them a promising antiviral agent [190]. Magnetite/silver core-shell NPs (∼20–80 nm in size) can deliver plasmid DNA with high cell penetration and efficient endosomal escape, overcoming some of the challenges associated with CRISPR/Cas9-based gene therapies (e.g. low cell delivery and unspecific targeting) [191]. Additionally, Ag NP-containing DNA-polymer complexes display enhanced stability and gene delivery efficiency with minimal cytotoxicity, highlighting their potential as robust carriers of therapeutic genes [191].

Ag NPs have shown considerable promise also for RNA delivery, particularly for cancer applications. Ag NPs modified with carbosilane dendrons and PEG efficiently delivered an siRNA against *MCL-1* (a gene belonging to the BCL-2 family that regulates apoptosis) to tumor cells (up to 90 % of cellular uptake) in order to induce cell death [192]. Moreover, DNA metallization with Ag NPs improves the stability and delivery efficiency of gene constructs, with minimal cytotoxicity, further supporting Ag NPs as robust gene delivery platforms in cancer treatment [193]. These findings underscore the effectiveness of Ag NPs in delivering RNA-based therapies to target and silence specific genes in cancer cells.

Bio-functionalization has significantly enhanced Ag NP specificity and efficiency for gene delivery. For example, PEG-stabilized chitosan-modified Ag NPs exhibit improved transfection efficiencies (up to 42 % in HeLa cells) and minimal toxicity, suggesting that they are safe, non-viral gene carriers. Similarly, Ag NP conjugation with the TAT peptide improves their cellular uptake and transfection efficiency, particularly in epidermal stem cells, suggesting possible regenerative medicine applications [194]. Additionally, Ag NPs offer a cost-effective alternative to gold NPs in gene therapy, particularly in plant gene delivery systems. In *Nicotiana tabacum* L. (tobacco), Ag NPs as gene carriers outperformed gold micro-carriers in terms of efficiency and cost-effectiveness, achieving higher transformation and regeneration efficiencies [195]. Besides surface modifications, the shape and size of Ag NPs, including spherical Ag NPs (2 nm), core-shell magnetite/Ag NPs (∼20–80 nm) [196], DNA-polymer-complexed Ag NPs (42.48 nm) [197] and rod-shaped Ag NPs (∼40 nm) [192], significantly influence gene delivery efficiency, stability and biocompatibility, emphasizing the versatility of Ag NPs for therapeutic and genetic engineering applications [198].

Many Ag NPs modified with molecules like folic acid, PEG, antibodies, or peptides have shown good results in lab and animal studies [2,18,21,199]. However, they are rarely used in actual clinical treatments yet. These modifications help the Ag NPs enter cells by binding to specific receptors. PEG helps the Ag NPs stay longer in the bloodstream. Targeting molecules guide them to cancer cells, lowering side effects on healthy cells. Inside the cell, Ag NPs release Ag^+^ ions, which create harmful ROS, damage mitochondria, and cause cell death. Despite these benefits, problems remain. Unintended effects on healthy cells can happen. Proteins in the blood can cover nanoparticles and reduce their targeting ability. Differences between production batches cause inconsistent results. More research is needed to make these nanoparticles consistent and safe for long-term use. So far, only a few have entered clinical trials; most are still being tested in the lab [2,18,21,199]. Table 6 shows examples of Ag NPs with different sizes and surface coatings used for delivering genes.

**Table 6:** Ag NPs for gene therapy applications.

Nanocarrier	Ag NP size & shape	Ag NP surface functionalization	Sequence type	Results	Ref
**PEG/CTS-g-PAAm@AgNPs/pDNA**	Spherical, 100–150 nm	Ag NPs with PEG and chitosan-grafted polyacrylamide (CTS-g-PAAm) to increase stability and biocompatibility	siRNA	- EV71 virus inhibition - Host cell infection inhibition - ROS production inhibition	[190]
**Magnetite/AgNPs-pDMAEMA-PEA-BUFII**	Core-shell, 462 nm	Magnetite core with Ag NP shell, poly(N,N-dimethylaminoethyl methacrylate)-polyethyleneamine (pDMAEMA-PEA) to promote DNA binding	Plasmid DNA	- DNA cargo loading: 16 % - DNA cargo release efficiency:8 % - Transfected Vero cell viability >95 %.	[191]
**AgNPs-PDMAEM-DNA-DAPI**	Spherical, 400 nm	Ag NPs with poly(N,N-dimethylaminoethyl methacrylate) (PDMAEM) for DNA binding and 4′,6-diamidino-2-phenylindole (DAPI) for fluorescence	Plasmid DNA	- Enhanced stability and luminescence	[200]
**PEG/CTS-g-PAAm@AgNP/pDNA/CTS-g-PAAm@AgNP/pDNA**	Spherical, 100–150 nm	Ag NPs with PEG and CTS-g-PAAm for stability and biocompatibility	Plasmid DNA	- PEG-Ag NP size: 38 ± 4 nm - Transfection efficiency: 42 ± 4 % (HeLa cells), 30 ± 3 % (A549) cells	[201]
**AgNPs-DNA**	Spherical, 100 nm	Ag NPs directly complexed with DNA	Plasmid DNA (pBI121)	- Higher transformation efficiency - Consumable costs reduction by 37.5-fold − 70.83 % of tobacco plant tissue regeneration	[202]
**AgNP-PEG-siMCL**	Spherical, 50–200 nm	PEGylated Ag NPs for stability and reduced toxicity	siRNA against *MCL-1*	- siRNA delivery: up to 90 % - Lower toxicity with higher PEGylation - Apoptosis induction	[192]
**AgNPs@PEI-TAT/DNA**	Spherical, 100–200 nm	Ag NPs with PEI-TAT to increase cell penetration	Plasmid DNA (EGFP)	- High transfection efficiency - Low cytotoxicity	[194]
**pDNA-AgNPs**	Spherical, 190 nm	Ag NPs directly complexed with DNA	Plasmid DNA	- High cell viability - Minimal cytotoxicity - Efficient gene delivery	[203]

## 7 Ag NPs for cancer immunotherapy and targeted treatment

**Fig. 13:**
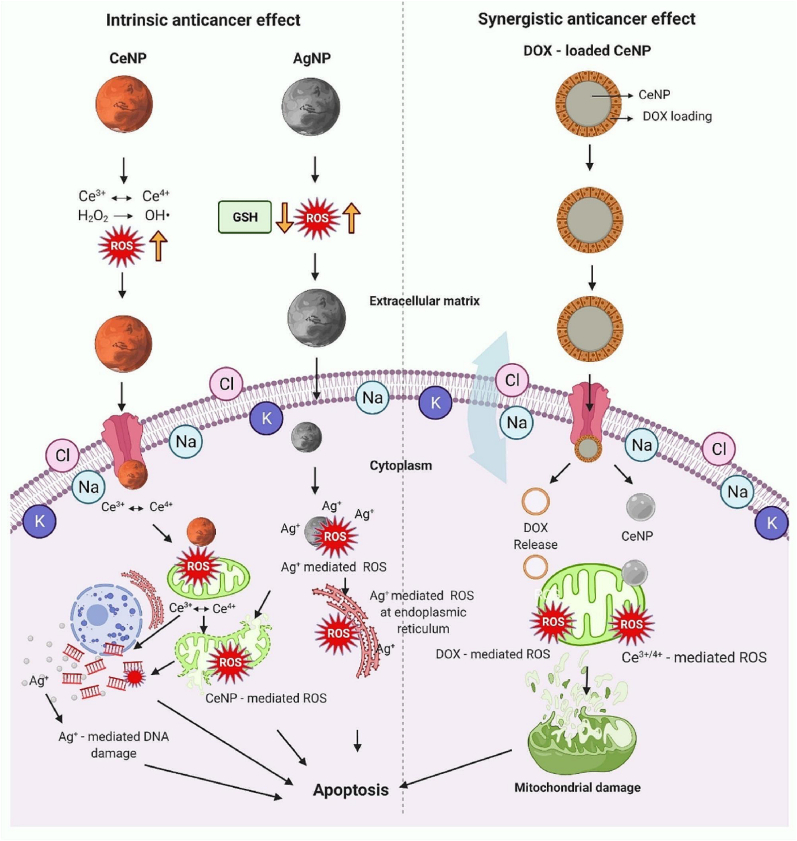
Intrinsic and synergistic anticancer effects of Ag NPs and cerium oxide Ce NPs in cancer treatment. Ag NPs induce apoptosis via ROS generation, leading to mitochondrial and DNA damage. Ce NPs facilitate cancer cell apoptosis through pH-dependent Ce^3+^ ↔ Ce^4+^ redox cycling that is increased when combined with doxorubicin (DOX). Ce NPs also exhibit selective protection of normal cells due to their unique prooxidant-antioxidant activity [206]. Reproduced with permission from Elsevier.

Ag NPs, typically ranging from 1 to 50 nm in size, are gaining significant attention in cancer therapeutics due to their unique physicochemical properties such as small size, large surface area, and high reactivity [204]. One key anticancer mechanism of Ag NPs is the generation of ROS, which induces oxidative stress that damages cancer cell DNA and mitochondria, leading to apoptosis. Additionally, Ag NPs can disrupt mitochondrial membrane potential, triggering programmed cell death, and interfere with cell cycle progression, thereby inhibiting cancer cell proliferation. Several studies also suggest that Ag NPs modulate key signaling pathways involved in tumor growth and metastasis, providing a multi-targeted approach to cancer treatment. To enhance therapeutic efficacy and targeting specificity, Ag NPs are often functionalized with anticancer drugs or biomolecules [205]. For example, conjugation with folic acid allows selective binding to cancer cells that overexpress folate receptors, improving targeted delivery and reducing toxicity. Furthermore, the ROS-mediated oxidative stress induced by Ag NPs contributes to DNA damage, cell cycle arrest, and tumor cell death through both apoptotic and non-apoptotic pathways, as illustrated in Fig. 13 [206]. These combined effects position Ag NPs as promising agents in cancer immunotherapy and targeted treatment strategies.

In the cell, Ag NP dissolution results in the release of Ag^+^ ions that can generate ROS. ROS production and the subsequent oxidative damage disrupt cellular processes, including membrane adhesion, mitochondrial function and signal transduction, ultimately causing mitochondrial dysfunction, protein damage, and DNA injury that trigger apoptosis or necrosis in cancer cells. The exact mechanisms of Ag NP toxicity remain debated. Some authors suggested that it is mediated by the released Ag^+^ ions, while others proposed that it is caused mainly by the NPs [[207], [208], [209]].

Recent evidence highlights that Ag NPs can initiate programmed cell death through multiple interconnected molecular cascades. Following cellular uptake, Ag NPs accumulate within the mitochondria and induce mitochondrial membrane depolarization via excessive generation of reactive oxygen species (ROS) [100]. This oxidative stress leads to the release of cytochrome *c* into the cytosol, which activates the intrinsic (mitochondria-mediated) apoptotic pathway through caspase-9 and downstream caspase-3 activation. Simultaneously, Ag NPs disrupt the balance of apoptotic regulators by increasing the expression of pro-apoptotic Bax and decreasing anti-apoptotic Bcl-2, thereby enhancing mitochondrial outer membrane permeabilization and committing cells to apoptosis [210].

In addition to ROS-mediated apoptosis, functionalized Ag NPs modulate multiple oncogenic signalling pathways. They inhibit the PI3K/AKT/mTOR pathway, a central regulator of cancer cell growth, metabolism, and survival [211]. Inhibition of this axis not only reduces proliferation but also promotes autophagic flux and apoptotic signalling. Ag NPs also enhance p53 phosphorylation, leading to cell cycle arrest and transcription of pro-apoptotic genes. Furthermore, activation of JNK and p38 MAPK pathways amplifies the oxidative stress response, contributing to stress-mediated apoptosis [212].

AgNPs can additionally affect cancer progression through non-apoptotic mechanisms, such as inducing cell cycle arrest, particularly in the sub-G1 phase, which reflects fragmented DNA and apoptotic induction [213]. Ag NP exposure has also been associated with inhibition of angiogenesis through downregulation of VEGF and HIF-1α, impairing neovascularization. This effect reduces nutrient supply to tumors and limits metastatic potential. *In vitro* and *in vivo* studies using cell lines such as A549 (lung), HepG2 (liver), MCF-7 and MDA-MB-231 (breast), and HeLa (cervix) confirm that Ag NPs reduce viability, increase ROS accumulation, inhibit migration, and trigger programmed cell death through the pathways described [211].

Ag NPs modulate the apoptotic pathways by inducing mitochondrial aggregation and ROS production, disrupting the mitochondrial membrane potential, and promoting the release of cytochrome *c*, which activates the intrinsic apoptosis pathway. Functionalized Ag NPs, especially those modified with phytochemicals or therapeutic ligands, have been reported to modulate the PI3K/AKT signaling pathway, a central axis regulating cancer cell proliferation, metabolism, and survival. The inhibition of this pathway disrupts downstream signaling cascades, thereby promoting the activation of pro-apoptotic factors, such as caspase-3 and Bax, and suppressing anti-apoptotic proteins like Bcl-2. This functional interference enhances apoptotic cell death and significantly reduces tumor cell viability, indicating a promising strategy for targeted cancer therapy [214]. Moreover, caffeine-derived Ag NPs induce G0/G1 phase arrest and apoptosis in A549 lung cancer cells [153]. Collectively, the ability of Ag NPs to generate ROS, disrupt mitochondrial function, and modulate apoptotic pathways underscores their potential as agents for targeted cancer therapy [215]. Biosynthetic Ag NPs limit the malignant behavior of gastric cancer cells and improve 5F's ability to reduce gastric cancer cell growth, migration, and invasion by inducing apoptosis and enhancing intracellular ROS generation [216].

Ag NPs (10–80 nm) anticancer properties are influenced by their size, shape, and surface functionalization. Small spherical Ag NPs (10–20 nm), synthesized using an *A. indica* leaf extract, show strong anticancer activity against HepG2 liver cancer cells via ROS-induced mitochondrial dysfunction and apoptosis [217]. Their small size favors their internalization into cells and DNA fragmentation, increasing cytotoxicity. Rod-shaped Ag NPs (50–80 nm), synthesized using CTAB, demonstrate higher uptake in A549 lung cancer cells, and consequently increased ROS production and mitochondrial depolarization, leading to elevated apoptosis rates [218].

Surface modifications further enhance Ag NP selectivity and therapeutic efficacy. PEG-coated Ag NPs (15–25 nm) display improved biocompatibility and circulation and lower systemic toxicity, while triggering ROS-mediated apoptosis. Citrate-capped Ag NPs (10–30 nm) boost intracellular ROS production, causing oxidative DNA damage in MCF-7 breast cancer cells [219]. Folic acid-conjugated Ag NPs (25 nm) selectively target folate receptor-overexpressing cancer cells, thus minimizing toxicity to normal tissues. Chitosan-functionalized Ag NPs (20 nm) show enhanced selectivity for colon cancer cells through electrostatic interactions, improving tumor penetration and apoptosis induction [220]. Glucose-functionalized Ag NPs (15 nm) exploit cancer cell high glucose uptake for selective cytotoxicity. These optimized physicochemical properties position Ag NPs as promising tools in targeted drug delivery, gene therapy and angiogenesis inhibition in order to improve cancer treatment with minimal side effects [221]. Table 7 lists the different anticancer mechanisms of Ag NPs synthesized from natural and chemical sources.

**Table 7:** Anticancer mechanisms of Ag NPs synthesized using natural and chemical sources.

Ag NP synthesis and characteristics	Ag NP surface functionalization	Anticancer mechanisms	Ref
Spherical Ag NPs (12 nm) synthesized from a *M. oleifera* extract	Functionalization with *M. oleifera* extract for enhanced biocompatibility and stability	Cytotoxicity and DNA damage in MCF-7 breast cancer cells, but not in human umbilical vein endothelial cells (HUVEC)	[222]
Spherical Ag NPs (8–48 nm)	Chitosan	Increased ROS production, which can cause oxidative stress, damaging cellular components and leading to cancer cell death	[223]
Spherical Ag NPs (10–30 nm) synthesized using an aqueous leaf extract of *Adenium obesum*	Functionalized with *A. obesum* leaf extract	Increased ROS production resulting in DNA damage, apoptosis, and autophagy in MCF-7 breast cancer cells	[224]
Spherical AgNPsCur and AgNPsCur@INH NPs (∼50 nm)	Curcumin (Cur) as reducing agent and stabilizing capping agent, forming a stable corona around the Ag NPs. Then, the surface was functionalized with isonicotinic acid hydrazide (INH)	Mitochondria mediate Ag NP toxicity, contributing to the selective death of LK-2 lung cancer cells compared with WI-38 normal lung fibroblasts	[225]
Spherical Ag NPs (14 nm) synthesized using a *Podophyllum hexandrum* Royle leaf extract	Functionalized with *P. hexandrum* leaf extract	Ag NPs selectively inhibit HeLa human cervical carcinoma cells through DNA damage and caspase-mediated cell death	[226]
Spherical Ag NPs (20–60 nm) using an *Olax scandens* leaf extract	Functionalized with *O. scandens* leaf extract	Ag NPs show anti-cancer activities in various cancer cell lines (A549 human lung cancer, B16 mouse melanoma, MCF-7 human breast cancer cells)	[227]
Spherical and cuboidal Ag NPs synthesized using plant extracts from *Gymnema sylvestre*, *M. oleifera* and *A. indica*	Functionalized with bioactive compounds from the plant extracts to improve biocompatibility and stability	Effects on *VEGF* and *CCND1* gene expression in A549 lung cancer cells	[204]
Spherical Ag NPs (75 nm) synthesized using caffeic acid	Functionalized with caffeic acid to improve stability and biocompatibility	In A549 lung cancer cells, cytotoxicity and cell cycle arrest	[228]
Ag NPs in two different sizes: 10 nm Citrate BioPure™ Silver and 75 nm Citrate BioPure™ Silver	Citrate-capped for stability	Effect on cell viability in precision cut lung slices from adult mice and in human lung fibroblasts (HFL-1); proinflammatory and immunomodulatory responses in HFL-1 cells	[229]

## 8 Ag NPs in chronic disease management

Due to their unique properties, Ag NPs can interact effectively with biological systems at the molecular and cellular levels, offering novel treatment options for neurological and cardiovascular disorders, albeit with potential risks [230].

Ag NPs could contribute to the management of neurodegenerative diseases, such as Alzheimer's disease and Parkinson's disease, due to their antioxidant, anti-inflammatory, and neuroprotective properties. Ag NPs enhance the activity of antioxidant enzymes, such as superoxide dismutase and catalase, thereby decreasing oxidative stress, a key factor in many neurodegenerative diseases. They also exhibit anti-inflammatory effects by inhibiting the activation of microglial cells and reducing the release of pro-inflammatory cytokines, thus promoting a neuroprotective environment. Importantly, Ag NPs can cross the BBB and reduce beta-amyloid aggregation, a hallmark of Alzheimer's disease. In Parkinson's disease, Ag NPs promote H_2_S production in microglial cells, reducing inflammation and supporting neuronal survival [231]. Nevertheless, concerns about Ag NP toxicity persist, and some studies suggest potential neuronal cell survival and synaptic activity impairment, highlighting the need of more research on surface functionalization and targeted delivery strategies to mitigate these risks.

In cardiovascular medicine, Ag NPs could be used to modulate angiogenesis and tissue repair, and also as antimicrobial coatings. Depending on their concentration, Ag NPs can inhibit or promote angiogenesis by modulating vascular endothelial growth factor (VEGF)-mediated signaling. At higher concentrations, Ag NPs block endothelial cell proliferation, which is useful in diabetic retinopathy and cancer. Conversely, at lower concentrations, they stimulate nitric oxide production, promoting angiogenesis, which is important in ischemic conditions [230]. Ag NPs also influence the vascular tone by modulating oxidative stress and nitric oxide pathways, promoting vasodilation at lower concentrations. However, they might impair endothelial function at higher concentrations [230]. Ag NPs are also widely used as antimicrobial coatings in cardiovascular devices to reduce the risk of infection associated with catheters and pacemakers [232]. However, cytotoxicity, ROS-induced endothelial damage and long-term bioaccumulation in organs require more investigations to fully determine Ag NP safety in cardiovascular applications.

*In vivo* studies in rodents have demonstrated that Ag NPs, when administered via inhalation, ingestion, or injection, accumulate in key organs such as the lungs, liver, kidneys, brain, and spleen [233]. Inhalation is a significant exposure route, with some studies reporting no adverse effects, while others observed pulmonary inflammation, altered lung function, and neurotoxicity [234]. For instance, short-term exposure to 20 nm Ag NPs altered brain gene expression, and longer exposures caused mild lung inflammation and silver accumulation in the brain and liver. Notably, Ag NPs have also been shown to cross the placenta in pregnant mice, raising concerns about fetal exposure. These findings underscore the need for standardized *in vivo* assessments to better evaluate the systemic toxicity and long-term safety of Ag NPs in biomedical applications [235].

**Fig. 14:**
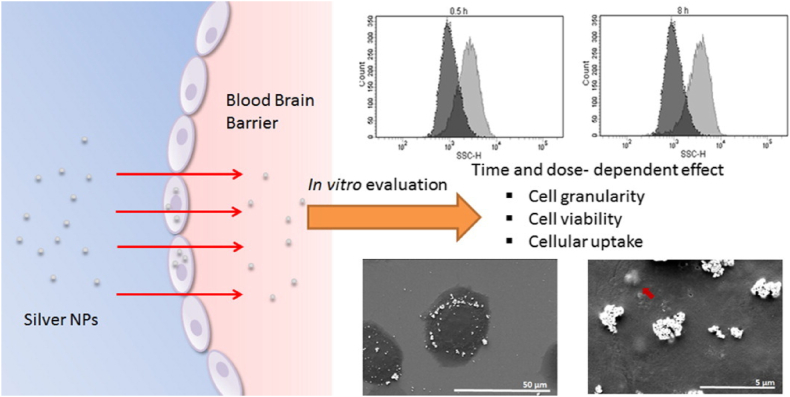
Graph illustrating the differential cytotoxic effects of Ag NPs on human endothelial cells. The study compares Ag NPs interactions with blood vessel endothelial cells (HUVEC and EA.hy926) and brain microvascular endothelial cells (HBEC5i), a model for the blood–brain barrier (BBB) [236]. Copyright, Elsevier.

Sokołowska et al. (2017) investigated Ag NP cytotoxic effects in blood vessel endothelial cells (HUVEC and EA.hy926) and in brain microvascular endothelial cells (HBEC5i), a BBB model [236]. They found that exposure to Ag NPs leads to NP agglomeration, resulting in compromised membrane integrity and decreased cell viability in all three cell types, but the effect was stronger in HUVEC and EA.hy926 cells than in HBEC5i cells (Fig. 14). These results provide critical insights into NP-induced BBB dysfunction and suggest that engineering safer Ag NPs with minimal neurotoxicity is essential for their biomedical applications [237].

## 9 Ag NP penetration in the skin

**Fig. 15:**
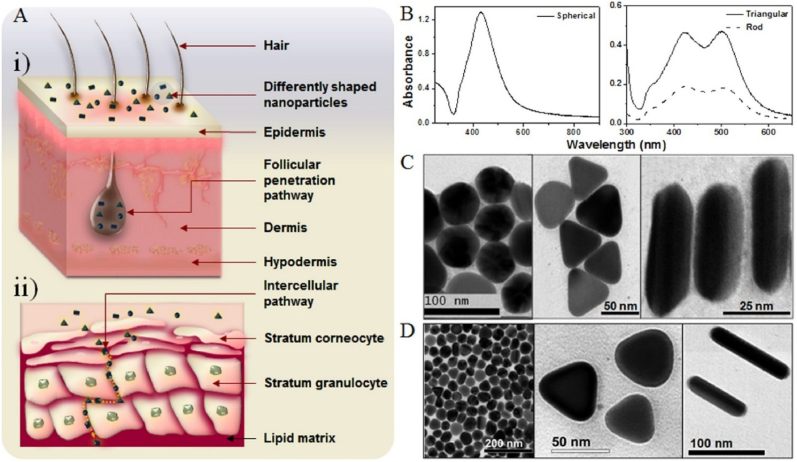
Penetration into the skin of Ag NP of different shapes. (A) Schematic representation of the i) follicular pathway and (ii) intercellular pathway. (B) Absorption spectra of chemically synthesized spherical, triangular, and rod-shaped Ag NPs, displaying characteristic absorbance peaks at specific wavelengths. (C) Transmission electron microscopy images illustrating the Ag NP morphology (spherical, triangular, and rod-shaped). (D) Transmission electron microscopy images confirming the presence of differently shaped Ag NPs in phosphate buffer (pH 7.0), the medium used in the donor compartment of the Franz cell, with particle dimensions consistent with their synthesized counterparts [238]. Open Access, Copyright Springer Nature.

Ag NP penetration in the skin can be modulated by tuning their size, shape and surface chemistry. Hair follicles are often considered the primary route for NP uptake (i.e. follicular pathway); however, a study challenged this assumption showing that interactions with the intercellular lipid matrix and the mechanical properties of NPs with different shapes also play a significant role [238]. The authors found that the Ag NP penetration rate into the skin was influenced by their shape (higher for rod-shaped and triangular vs spherical NPs). As the hair follicle opening (∼26 μm) is much larger than the Ag NP size (∼45–50 nm), similar penetration of the three Ag NP shapes would be expected if the follicular pathway were the key player. However, smaller Ag NPs may more easily diffuse through the stratum corneum (i.e. the intercellular pathway), and shape variations influence interactions with the skin layers and bacterial cells (Fig. 15). These findings have major implications when Ag NPs are used for topical antibacterial applications.

Indeed, using *in vitro* and *in vivo* models, they demonstrated that differently shaped Ag NPs exhibit variations in penetration rates, diffusion coefficients, and penetration depth, particularly through the intercellular pathway [238]. On the basis of their penetration and shape-dependent bactericidal activity, triangular NPs could be an ideal candidate for topical applications, compared with rod-shaped and spherical Ag NPs [166]. The variations in penetration rates suggest additional mechanisms at play and indicate the need for high-resolution imaging (e.g. transmission electron microscopy) studies to clarify the penetration mechanisms. They also highlight the importance of considering the NP shape when designing topical antimicrobial formulations with optimized efficacy and minimal systemic toxicity [239].

## 10 Clinical translation and regulatory challenges

Despite the promising applications of Ag NPs in diagnostics, drug delivery, and antimicrobial treatments, several critical limitations hinder their successful translation into clinical practice. One major challenge is the scalability and reproducibility of Ag NP synthesis [240]. Techniques such as chemical reduction, seed-mediated growth, and green synthesis often result in high batch-to-batch variability due to their sensitivity to pH, temperature, and precursor concentration fluctuations [241]. Even minor deviations during synthesis can significantly alter particle shape and size distribution, ultimately affecting their surface plasmon resonance (SPR) behavior and biological activity [242]. Moreover, although numerous *in vitro* studies report the high efficacy of Ag NPs against cancer cells, these findings rarely translate into clinical success due to the complexity of human biology and insufficient predictive power of preclinical models [243]. Furthermore, concerns regarding the accumulation of Ag NPs in major organs (e.g., liver, spleen, and kidneys), the generation of reactive oxygen species (ROS), and potential genotoxic effects have led regulatory agencies such as the FDA and EMA to advocate for more stringent standardization protocols and comprehensive toxicity profiling to ensure safety and reproducibility [244].

Most research on Ag NPs has been confined to preclinical studies, and only a small fraction has advanced to human trials. Key barriers to their clinical translation include the lack of standardized production protocols, reproducibility of results across biological systems, and optimization of delivery methods. Regulatory agencies (FDA and EMA), require extensive safety and efficacy data before approval for medical use. The regulatory approval process is also complicated by the fact that Ag NP biological behavior is significantly influenced by their unique physicochemical properties (e.g. size, shape and surface modifications) and by the heterogeneity of Ag NP formulations that necessitates robust characterization protocols to ensure consistency [245] and predictability in clinical outcomes [246]. Moreover, the marketing of Ag NP-based therapies requires overcoming issues related to scalability, cost-effectiveness and quality control after having demonstrated the long-term benefits of these therapies [247,248].

Nanocrystalline silver-based products, such as Acticoat®, have successfully transitioned from the lab to clinical practice, particularly in burn and wound management. Silver nitrate solution, silver sulfadiazine cream, and Acticoat® were compared against 11 clinical isolates of antibiotic-resistant bacteria, revealing that Acticoat® demonstrated the most rapid and effective bactericidal activity [249]. Acticoat® was shown to release Ag^+^ ions more efficiently than other silver formulations, resulting in a faster bacterial kill rate and enhanced antimicrobial performance [250]. In another study, Thomas et al. (2003a, 2003b) [251] showed that Acticoat® had superior antimicrobial activity across a broader spectrum of bacteria and fungi, including MRSA. Despite these promising results, clinical translation faces challenges, such as the need for extensive safety and efficacy evaluations by regulatory bodies like the FDA. Concerns about the cytotoxicity of Ag^+^ ions, especially towards keratinocytes and fibroblasts, underscore the need for careful formulation assessments. Despite these challenges, Acticoat® exemplifies the therapeutic potential of Ag NPs, showing that with robust preclinical and clinical data, silver nanotechnology can become a viable clinical product [252].

To improve biocompatibility and therapeutic outcomes, strategies such as functionalizing Ag NPs with biocompatible polymers or targeting ligands have been proposed to enhance stability, specificity, and cellular uptake. Controlled-release formulations and dose-optimization techniques can further reduce adverse effects while maintaining efficacy. As regulatory frameworks evolve, it is essential that they integrate emerging data on Ag NP interactions with biological systems to inform evidence-based guidelines for their safe use. Advanced *in vitro* and *in vivo* models, along with computational toxicology, will play a critical role in predicting human responses and refining safety evaluations. Long-term studies are particularly necessary to assess the cumulative, generational, and environmental impacts of Ag NP exposure, ensuring both safety and efficacy [253]. Balancing the benefits and risks of Ag NPs requires a proactive and ethically grounded regulatory framework. While their biomedical potential is significant, concerns over long-term toxicity, environmental accumulation, and unclear exposure limits demand precautionary oversight [254]. Regulatory agencies should adopt adaptive, evidence-based policies that evolve with scientific understanding. Comprehensive life-cycle assessments, transparent risk-benefit analyses, and ethical guidelines; particularly in clinical trials and patient consent are essential [255]. Ensuring safe, sustainable, and socially responsible development of Ag NPs is critical to realizing their therapeutic potential without compromising human or environmental health. Table 8 lists the applications and current clinical research on Ag NPs and key challenges.

**Table 8:** Ag NPs in the clinic and key challenges.

Category	Details	Ag NP shape and size	Ag NP surface functionalization	Examples	Key challenges	Ref
Clinical applications	Wound healing, antimicrobial coatings, cancer therapy	Spherical, 10–100 nm,	Functionalized with polyvinylpyrrolidone (PVP), citrate or chitosan for enhanced stability and bioactivity	Ag NP-based dressings (e.g. Acticoat), experimental cancer therapies	Limited data on long-term efficacy	[256]
Ongoing clinical trials	Studies on Ag NP safety, dose and efficacy in humans	Spherical, 12 nm	Stabilized with PVP and iodine-125	Trials in patients with burns, oral health	Small sample sizes, limited patient population diversity	[257]
Regulatory approval	Products cleared for specific applications (e.g. wound care)	Spherical, 50 nm	PVP-coated Ag-NPs using alveolar macrophages	FDA-cleared Ag NP dressings	Inconsistent regulatory requirements around the world	[258]
Obstacles to regulatory approval	Safety concerns, reproducibility, scalability	Not specified	Not specified	Toxicity (organ accumulation), NP synthesis variations	Absence of harmonized testing standards	[259]
Strategies for challenge overcoming	Standardization, advanced toxicological tests, targeted delivery systems	Not specified	Not specified	Functionalized Ag NPs, computational modeling, rigorous long-term safety studies	High cost of research and regulatory compliance	[260]

## 11 Conflicting mechanisms and ongoing controversies in Ag NPs toxicity

Despite increasing interest in the biomedical applications of Ag NPs, significant controversy persists regarding their toxicity mechanisms. A central question is whether cytotoxic and genotoxic effects are primarily due to the Ag NPs themselves or to (Ag^+^) released during dissolution. Some studies suggest that toxicity is largely ion-mediated, as Ag^+^ disrupts redox balance, mitochondrial function, and membrane integrity [261]. In contrast, other researchers report that intact Ag NPs can penetrate cells, induce oxidative stress, and interact with intracellular components independently of ion release [262].

This discrepancy is influenced by experimental conditions such as pH, ionic strength, and the presence of biomolecules, all of which modulate Ag^+^ release. Smaller particles, due to their higher surface-area-to-volume ratios, dissolve more readily, increasing ion-mediated toxicity. Conversely, surface functionalization (e.g., with plant polyphenols or polyethylene glycol [PEG]) can stabilize particles and reduce dissolution [263]. Supporting the ion-driven mechanism, studies using ion chelators or Ag^+^-specific inhibitors have shown significantly reduced toxicity. However, other studies provide evidence for the cellular uptake of intact Ag NPs, causing mitochondrial swelling and lysosomal disruption, indicating direct nanoparticle-induced damage [264]. Thus, both particulate and ionic forms contribute to Ag NP toxicity in a context-dependent manner. Understanding this dual behaviour is essential for the rational design and safety assessment of Ag NPs in medical applications.

In addition to these mechanisms, Ag NPs exhibit bioaccumulation and organ-specific toxicity. Their accumulation is size-dependent, with smaller nanoparticles (<10 nm) demonstrating higher permeability across biological membranes and greater retention in the liver, kidneys, spleen, and brain. These organs are particularly susceptible to Ag NP-induced oxidative stress, inflammation, and tissue damage due to their roles in detoxification and filtration [265]. However, Larger particles tend to accumulate less and are cleared more efficiently [100].

**Fig. 16:**
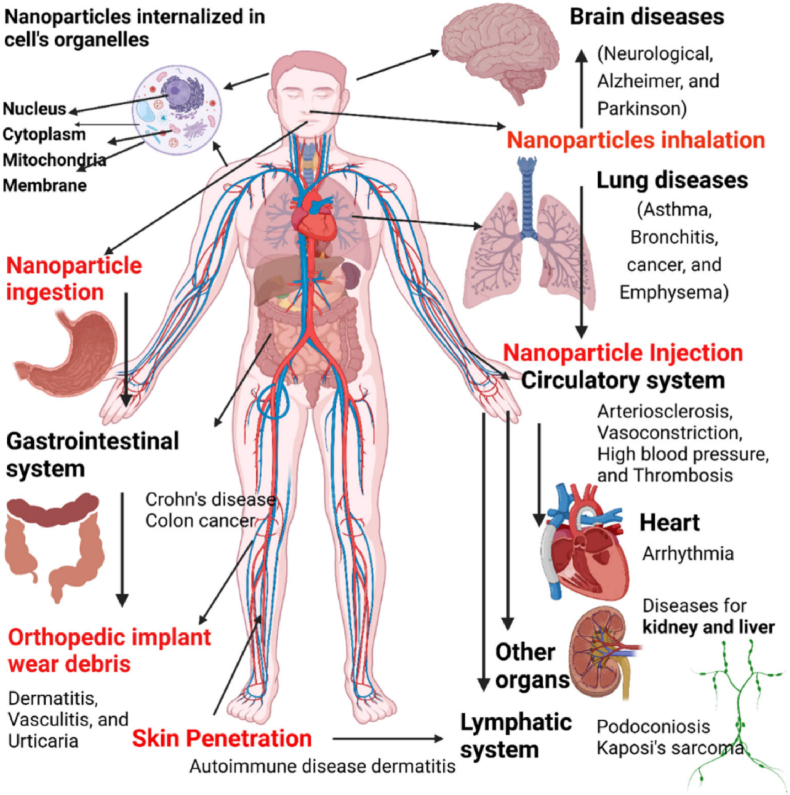
Routes of Ag NPs entry into the body including inhalation, dermal absorption, and ingestion and the associated health risks and diseases caused by exposure [266]. Open Access, MDPI.

Fig. 16 shows potential health risks associated with Ag NP exposure, outlining primary entry routes such as inhalation, dermal absorption, and ingestion [266]. Exposure to Ag NPs has been linked to neurological diseases (e.g., Parkinson, Alzheimer), respiratory disorders (asthma, bronchitis, emphysema, lung cancer), cardiovascular problems (atherosclerosis, arrhythmia, thrombosis, hypertension), and dermatological conditions (irritation, dermatitis, urticaria). Once internalized, Ag NPs activate the IKK/NF-κB signaling pathway, leading to excessive ROS generation. This cascade results in cytoskeletal disruption, DNA damage, and impaired DNA repair. Even at low doses, Ag NPs have been shown to changes in metabolism, modulate oxidative stress-related gene expression, and interfere with the cell cycle, particularly in human skin fibroblasts. These findings highlight the complex and potent cytotoxic effects of Ag NPs, underscoring the need for thorough toxicological evaluation [267].

Efforts to mitigate toxicity focus on optimizing physicochemical properties such as size, surface chemistry, and morphology. Recent studies demonstrate that surface modification (e.g., PEGylation or phytochemical capping) and precise size control can reduce toxicity while maintaining therapeutic efficacy. Additionally, green synthesis methods using natural stabilizers improve biocompatibility without compromising biomedical functionality [267].

## 12 Future directions and challenges

Ag NPs offer promising avenues for advancing medical treatments due to their tunable size, shape, and surface properties, enabling applications in drug delivery, immunotherapy, tissue regeneration, and chronic disease management. However, challenges such as high production costs, scalability, safety concerns, and regulatory barriers must be addressed for successful clinical translation [268]. To bridge the gap between research and clinical application, future efforts should prioritize the standardization of synthesis and functionalization protocols, as well as long-term *in vivo* studies on biodistribution, clearance, and potential toxicity. A deeper understanding of Ag NP interactions with the immune system and microbiome is also essential. Among all physicochemical features, size, shape, and surface functionalization are the most critical, as they influence nanoparticle stability, biocompatibility, and therapeutic precision [157]. Tailored surface engineering: especially with targeting ligands and biocompatible coatings may ultimately determine the success of Ag NP-based therapies in personalized medicine. The next paragraphs outline key future directions and challenges in Ag NP clinical applications and some potential solutions.

(1) **Medical applications:** Ag NPs hold immense promises for many medical applications, particularly for managing diseases where conventional therapies give limited results. These applications include precision drug delivery, regenerative medicine, cancer immunotherapy and antimicrobial treatments.
(2) **Diabetes management:** For patients with diabetes, Ag NPs could be exploited in controlled delivery systems for insulin or other antidiabetic agents, providing more targeted and effective therapy. Additionally, Ag NP antioxidant properties could mitigate oxidative stress, a major contributor to diabetes complications (neuropathy, retinopathy, and nephropathy) [269]. Therefore, Ag NPs could enhance the patients' quality of life and reduce the risk of long-term diabetes-related health issues.
(3) **Autoimmune diseases:** Ag NPs offer a novel approach to managing autoimmune diseases, such as rheumatoid arthritis and multiple sclerosis, by acting as targeted immunomodulators. Their ability to modulate the immune response and to reduce chronic inflammation can decrease the need of broad-spectrum immune suppressors. This targeted action may help to limit disease flare-ups and tissue damage, providing hope for patients who currently have limited treatment options [270].
(4) **Age-related conditions:** Ag NPs could offer valuable solutions for age-related conditions, such as Alzheimer's disease, cardiovascular degeneration and dermal atrophy. Their antioxidant effects and ability to stimulate collagen synthesis and tissue regeneration may provide new treatment avenues for age-associated ailments, enhancing the patients' quality of life and improving their long-term health outcomes [271].
(5) **Multidisciplinary research:** The clinical application of Ag NPs could be significantly accelerated through multidisciplinary research projects that involve nanotechnology, regenerative medicine, and systems biology scientists. Such collaborations would help to develop more precise and effective Ag NP-based treatments for complex diseases that require multifaceted approaches and to offer customized solutions for patients with diverse needs [272].
(6) **Production costs and sustainability:** The current Ag NP production methods are often expensive and environmentally unsustainable. To make Ag NPs more accessible for medical applications, cost-effective and scalable green synthesis methods must be developed. Plant extract-based and microbial fermentation approaches could reduce both production costs and their environmental impact, making of Ag NPs a more viable option for widespread use in healthcare [273].
(7) **Regulatory and safety concerns:** Despite Ag NP potential, regulatory challenges and safety concerns need to be addressed. Standardized production protocols and comprehensive safety evaluations are essential to meet regulatory standards and ensure safe use in humans. In addition, Ag NP potential toxicity, particularly in the case of long-term exposure, calls for rigorous toxicity studies and the development of safe formulations with minimal side effects. Regulatory bodies must work with researchers to establish clear safety guidelines, including the assessment of Ag NP biodistribution and long-term biocompatibility.
(8) **Standardization and quality controls:** The many Ag NP synthesis methods can lead to inconsistencies in particle size, shape and surface functionality, which may affect their therapeutic efficacy and safety. The size and morphology of Ag NPs depend on interactions between Ag NPs and phytochemical capping agents. Common phytochemicals such as amides, flavonoids, and peptides are involved in Ag NP synthesis, but their precise roles and interactions remain unclear [274]. Controlling size and shape is challenging due to the complex nature of biological reducing agents and factors like pH, temperature, extract concentration, and reaction duration, which significantly influence nanoparticle characteristics [106]. Scaling up green synthesis methods is limited by the seasonal availability of biological materials and the difficulty of maintaining consistent reaction conditions on large scale [275]. Additionally, purifying biosynthesized nanoparticles is challenging, as residual bio-organic compounds on Ag NPs surfaces may interfere with applications that require strict surface control and high chemical purity [276]. Standardized quality control protocols must be put in place to ensure the uniformity and reproducibility of Ag NP formulations. Real-time *in-situ* analyses may help to reduce variations, by facilitating the synthesis process monitoring. This will enhance Ag NP acceptance in clinical settings and support their approval [277].

## 13 Conclusion

Silver nanoparticles (Ag NPs) offer promising opportunities in modern medicine due to their tunable size, shape, and surface functionalities. These features enable a broad range of therapeutic applications. This review highlights the role of Ag NPs in addressing key healthcare challenges such as antimicrobial resistance, targeted drug delivery, cancer therapy, and regenerative medicine. Advances in green synthesis and surface modifications have improved their biocompatibility, stability, and therapeutic efficiency while promoting sustainability. Additionally, emerging applications in neurological and cardiovascular disease management underscore their clinical potential. However, clinical translation of Ag NPs remains limited. Challenges include toxicity concerns, poor scalability, batch-to-batch variability, and complex regulatory approval processes. Despite widespread use in preclinical research, few Ag NP formulations have reached clinical trials, with the need for validation. Overcoming these barriers requires standardized production, long-term safety studies, and regulatory alignment. While Ag NPs represent significant advancement, their integration into routine clinical practice must be guided by safety evaluation. Continued interdisciplinary efforts may support the development of precision medicine. However, claims of a major breakthrough should be based on solid evidence and consider existing limitations.

## CRediT authorship contribution statement

**Ibtissam Laib:** Writing – review & editing, Writing – original draft, Visualization, Validation, Supervision, Software, Resources, Methodology, Investigation, Funding acquisition, Formal analysis, Data curation, Conceptualization. **Noura Gheraissa:** Writing – review & editing, Writing – original draft, Visualization, Validation, Supervision, Investigation, Funding acquisition. **Abir Benaissa:** Writing – review & editing, Writing – original draft, Visualization, Validation, Supervision, Resources, Data curation, Conceptualization. **Latra Benkhira:** Writing – original draft, Visualization, Formal analysis, Data curation, Conceptualization. **Manel Azzi:** Writing – review & editing, Writing – original draft, Visualization, Formal analysis, Data curation, Conceptualization. **Yousef Benaissa:** Writing – original draft, Visualization, Funding acquisition, Formal analysis, Data curation, Conceptualization. **Ahmed G. Abdelaziz:** Writing – review & editing, Writing – original draft, Visualization, Validation, Formal analysis, Data curation, Conceptualization. **Furong Tian:** Writing – review & editing, Visualization, Validation, Supervision, Data curation, Conceptualization. **Maureen Walsh:** Writing – review & editing, Writing – original draft, Visualization, Validation, Formal analysis, Data curation. **Mikhael Bechelany:** Project administration, Funding acquisition, Formal analysis, Data curation, Conceptualization. **Ahmed Barhoum:** Writing – review & editing, Validation, Supervision, Software, Resources, Project administration, Methodology, Investigation, Funding acquisition, Formal analysis, Data curation, Conceptualization.

## Declaration of competing interest

The authors declare that they have no known competing financial interests or personal relationships that could have appeared to influence the work reported in this paper.

## Data Availability

Data will be made available on request.
